# A Computational Model for Spatial Navigation Based on Reference Frames in the Hippocampus, Retrosplenial Cortex, and Posterior Parietal Cortex

**DOI:** 10.3389/fnbot.2017.00004

**Published:** 2017-02-07

**Authors:** Timo Oess, Jeffrey L. Krichmar, Florian Röhrbein

**Affiliations:** ^1^Department of Informatics, Technical University of Munich, Garching, Germany; ^2^Cognitive Anteater Robotics Laboratory, Department of Cognitive Sciences, University of California Irvine, Irvine, CA, USA

**Keywords:** spatial navigation, frames of reference, retrosplenial cortex, posterior parietal cortex, hippocampus, computational model

## Abstract

Behavioral studies for humans, monkeys, and rats have shown that, while traversing an environment, these mammals tend to use different frames of reference and frequently switch between them. These frames represent allocentric, egocentric, or route-centric views of the environment. However, combinations of either of them are often deployed. Neurophysiological studies on rats have indicated that the hippocampus, the retrosplenial cortex, and the posterior parietal cortex contribute to the formation of these frames and mediate the transformation between those. In this paper, we construct a computational model of the posterior parietal cortex and the retrosplenial cortex for spatial navigation. We demonstrate how the transformation of reference frames could be realized in the brain and suggest how different brain areas might use these reference frames to form navigational strategies and predict under what conditions an animal might use a specific type of reference frame. Our simulated navigation experiments demonstrate that the model’s results closely resemble behavioral findings in humans and rats. These results suggest that navigation strategies may depend on the animal’s reliance in a particular reference frame and shows how low confidence in a reference frame can lead to fluid adaptation and deployment of alternative navigation strategies. Because of its flexibility, our biologically inspired navigation system may be applied to autonomous robots.

## 1 Introduction

The task of orienting oneself in an unknown environment and hence being able to find a route from one place to another seems quite obvious at first glance. However, if one wants to model that behavior artificially, there are significant problems to overcome: (1) How can an agent relate the perceptual information to previously stored memory of the same environment? (2) How can an agent retrieve stored spatial memory and transform it, such that it is useful in the current context? (3) How does an agent combine the various sensory information to infer its current position and plan a path through space?

We intend to answer these questions with our cognitive model of different brain regions by conducting several experiments in which our agent needs to combine different sensory information, relate it to its current position and retrieve stored spatial memory in order to successfully navigate.

Based on behavioral findings in rodents, Tolman (1948) introduced the concept of a *cognitive map* in 1948. Since then, the underlying neural principles of constructing such a cognitive map have been attributed to neuron types (such as place, head direction (HD) and grid cells) in different brain regions and are under extensive investigations.

With their publication, O’Keefe and Nadel (1978) set the basis for modern neurological exploration of brain areas that are thought to be responsible for navigation. They reported that the rat’s *hippocampus* constructs the previously proposed cognitive map and thereby is crucial for navigational capabilities. Many biologically inspired navigation models have been deployed on robots (Arleo and Gerstner, 2000; Krichmar et al., 2005; Strösslin et al., 2005; Barrera and Weitzenfeld, 2008; Milford and Wyeth, 2008; Erdem et al., 2015). Some of these biologically inspired models can outperform conventional engineering and robotics localization and mapping algorithms, like *Extended Kalman-Filters* (Dissanayake et al., 2001; Huang and Dissanayake, 2007) or *Particle Filters* (Montemerlo and Thrun, 2007) under certain scenarios (Prasser et al., 2006).

Several neurophysiological and behavioral studies have tested a subject’s ability to construct a cognitive map of the environment and, based on that, their navigational behavior (Zinyuk et al., 2000; Basten et al., 2012; Fouquet et al., 2013). Data from these studies facilitated researchers to build simulations of different granularity (Worden, 1992; Redish and Touretzky, 1997; Wiener and Mallot, 2003; Madl et al., 2015). Those range from the precise simulation and measuring of single neurons to the replication of behavioral responses. The simulations built on that data are crucial for our understanding of the underlying principles without the need to conduct additional studies with living creatures.

However, how the cognitive map is utilized and manipulated, which is required for successful navigation, is still not fully understood. To date, there exists only a few simulations that model the manipulation of spatial representations within a cognitive map (Byrne et al., 2007; Meilinger, 2008; Wolbers and Wiener, 2014).

One crucial concept for navigating is the *spatial frames of references*. A spatial reference frame is a representation, i.e., a coordinate system of locations or orientations of entities in an environment. Recent studies suggest that there are three major frames of references involved in spatial navigation, namely *egocentric, allocentric*, and *route-centric* (Nitz, 2012; de Condappa and Wiener, 2016).

Each frame can be defined by fixing the origin and orientation of the coordinate system to a specific entity within, or independently of, the environment. This might be the viewer himself or a particular landmark. In several behavioral and/or neurophysiological studies, researchers reported that humans as well as animals show typical behavior and/or neuronal responses, which indicate that the test subject uses different frames of references in order to navigate in the environment (Committeri et al., 2004; Galati et al., 2010; Nitz, 2012; Alexander and Nitz, 2015).

The construction of these frames is assumed to take place in different regions of the brain. Some frames exclusively exist in one brain region whereas others are constructed in one region but maintained over several others. Thereby, it is assumed that frames can exist in multiple regions and that there are smooth transformations from one region to another (Galati et al., 2010). The *hippocampus* (HPC), the *retrosplenial cortex* (RSC), and the *posterior parietal cortex* (PPC) are three main regions for the construction and maintenance of spatial reference frames (Committeri et al., 2004). More background on how these brain regions contribute to different frames of reference can be found in Section S1 in Supplementary Material.

Few computational models have investigated those areas in regard to their ability to construct spatial reference frames and to transfer between them. The work of Byrne et al. (2007) is worth mentioning here since they built a comprehensive model that includes the encoding of long-term and short-term memory as well as the transformation between these. Their primary focus is on the exact replication of the neural mechanisms for the transformation and retrieval of spatial memory. Sensory input is used to drive the egocentric–allocentric transformation and to build what they call the boundary vector cells (BVC).

In the present work, we introduce a model to investigate how different frames of reference are utilized during spatial navigation. Rather than constructing a neural network model of navigation, we construct an algorithmic description of the type of information processed by different brain areas important for navigation. We assume that the (1) HPC carries an allocentric frame of reference. (2) The PPC contains an egocentric and route-centric frame of reference. (3) The RSC, which has access to all three frames of reference, selects the most reliable frame of reference to be utilized for navigation. Landmark information (including connections between them) is provided to the model as input. Each area calculates a goal direction and feeds it to the RSC, where a corresponding confidence level is computed. The RSC decides which frame of reference to deploy based on that confidence. The model is able to replicate the navigation behavior from several animal and human studies. Our simulations suggest that navigation strategies depend on the agent’s confidence in a particular reference frame and that the decision to rely on such information can fluidly change depending on sensory inputs. In the remainder of the paper, we explain the different brain regions and their hypothesized contribution to the principle of reference frames in detail and show neurophysiological and behavioral findings that lead to these hypotheses.

Afterward, the model is described comprehensively and its biological plausibility is discussed. In order to validate the model, we conduct several different experiments that replicate human and animal spatial navigation studies.

## 2 Materials and Methods

**Figure 1:**
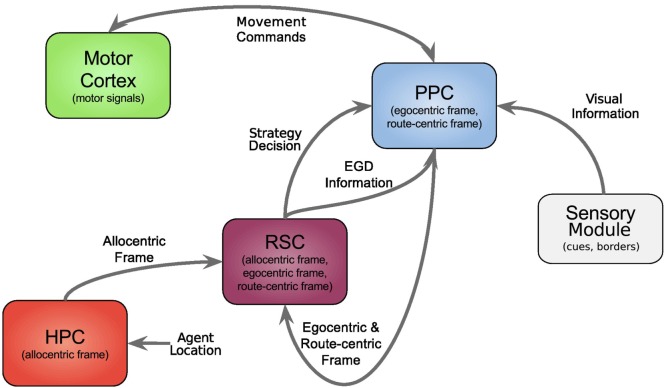
**Architecture overview**. Colored boxes show modeled brain regions with subscriptions to identify what information is comprised. Arrows indicating information flow direction and captions the type of conveyed data.

In this section, we first give an overview of our model followed by a comprehensive explanation of all modules, and the data flow is depicted in Figure 1, each modeling a specific part of the brain and their relevant connections.

### 2.1 Model Overview

A neurally inspired computational model is presented that shows how different spatial reference frames can be combined for effective navigation. An important goal for this model is that it follows neurobiological findings and assumptions. However, it is not the intention to model each brain region exactly and comprehensively at the cellular layer. Rather, the focus is on simulating the connectivity and function of the brain regions, as well as the information flow between them, i.e., what area sends what kind of information to which area. Thereby, we intend to get a better insight in how spatial frame transformation is realized on a coarse level and how it works for spatial navigation in different environments, without paying much attention on how the data are acquired biologically. Although, we simulate some specific neural activity when necessary in order to reproduce key findings. We provide a background and some examples for behavioral experiments in Section S1 in Supplementary Material.

All modeled areas of the brain and their proposed connections are illustrated in Figure 1. The boxes are colored uniquely and we keep referring to those colors throughout this work when showing a specific area.

The model is divided into several modules, each modeling a specific part of the brain, and the available information for each module is constrained according to corresponding neurological findings.

The agent’s location, which in the current implementation is directly obtained from the environment is fed into the HPC module (red box, bottom left). The HPC is modeled as a constructed cognitive map of the environment and keeps track of the agent, landmarks, and goal locations. Using this information, a goal vector from the agent to the goal can be determined. The goal direction is encoded in an allocentric manner and thereby constructs the allocentric frame which is then fed into the RSC (purple box). This module utilizes head direction information to transform the received allocentric frame to an egocentric representation of the goal direction, the egocentric frame. An egocentric frame is a representation of objects with the agent as the center or origin of the frame, whereas the allocentric frame of reference encodes objects independently of the head direction of the agent using one object as the origin. In this case, the egocentric frame encodes the goal direction by the degrees the agent has to turn in order to face the goal. The RSC also maintains levels of confidence for each frame. These levels describe how certain the agent is about the correctness of a frame that is to what extent can this frame help the agent to progress on its trajectory to the goal. Several information sources (i.e., in accordance with other frames about the goal direction, achieved progress using this frame, progress toward the goal applying this frame) are taken into account to determine a frame confidence value. Based on these values, the RSC decides which frame should be applied consecutively by drawing a frame from a probability distribution built on the confidence values and sends this decision as well as the egocentric frame to the PPC (blue box). A sensory module endows the PPC with information about landmarks and borders of the environment. Given that information, the egocentric frame and the previously stored route-centric frame, the PPC calculates different navigation strategies, each based on one frame of reference. The egocentric strategy involves the egocentric frame of reference and information about landmarks in order to determine that landmark for which the agent’s egocentric direction is closest to the goal direction and subsequently generate movement signals to proceed to that landmark. The route-centric strategy is based on a previously stored sequence of either motor commands with corresponding choice points or cues that has to be followed in order to reach the goal. The movement signals are then either directly applied or calculated based on the next landmark to visit. The allocentric navigation strategy only involves the overall direction to the goal. Note here, it is impossible to utilize solely an allocentric goal direction (AGD) for navigation, since the agent has to relate allocentric information to its current perspective. In order to navigate using an allocentric frame, it has to be related to the agent’s perspective and therefore translated into an egocentric frame. However, when applying the allocentric strategy, the PPC calculates a movement signal based on a goal direction, which was directly translated from the allocentric frame without considering additional information about landmarks. Finally, the movement signal is sent to the motor cortex (green box) where it is translated to motor commands for the agent.

In the following, we describe every module in detail and provide neurological data that support our module design choices.

### 2.2 Hippocampus

Due to its involvement in several major brain functions as memory consolidation, transformation from short-term memory to long-term memory and vice versa and its important role in spatial navigation and localization, the HPC has been greatly investigated over the last several decades. O’Keefe and Nadel (1978) stated in their work that the HPC established a cognitive map with the help of place cells. The cognitive map played a key role in every aspect of spatial navigation in larger environments. HPC lesions in rodents led to impairments in retrieval of spatial memory and as a consequence failure in navigational task (Morris et al., 1982; Dumont et al., 2015).

The most important feature of the HPC for our work is the encoded cognitive map. This map is enriched with numerous meta information for each landmark, path, cue, or object that can be used for action planning in the environment (i.e., moving one’s hand to grasp an object or calculating the shortest path from one place to another). Meta data for each entity can comprise many different types of information as color, shape, smell, appearance, location, and spatial relations to other objects (encoded in vectors). Since the beginning of navigation research in rodents, it is assumed that rodents utilize the cognitive map in the HPC to retrieve and process such vectors. They use corresponding “vector-based calculations” to plan a route from one location to another (O’Keefe and Nadel, 1978; Gallistel, 1990; McNaughton et al., 1996). These calculations facilitate the agent to determine the allocentric direction to a goal from its current position. Using the allocentric direction and the relations between objects in the environment, an allocentric frame is constructed and maintained in the HPC.

**Figure 2:**
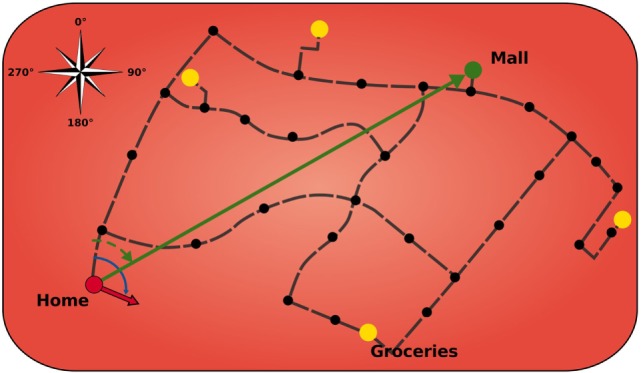
**Model of hippocampus**. Black dots indicate landmarks. Yellow circles display important locations that can serve as goals. Green arrow is the allocentric goal direction. Red arrow is the current head direction of the agent. Black dashed lines depict connection vectors with the allocentric direction and distance of the adjacent landmarks.

Similar to its biological counterpart, the modeled HPC is comprised of a topological map of the environment that stores salient landmarks and connections, including meta information such as direction and distance (Figure 2). The HPC module is designed to keep track of the agent’s location in the environment and to calculate paths to a goal using landmarks and vector calculations. The black dots indicate that these landmarks can be a crossing or a structure of the road and that helps to orientate the agent. Furthermore, the HPC facilitates the agent with a sense of an allocentric direction of the goal from the agent’s current location.

In the current model, landmarks are in Cartesian coordinates that are encoded by place cells, so that each time the agent approaches a landmark the corresponding place cell gets activated. Rather than using the population of place cells to encode a location, we used a Winner Take All principle, in which only the place cell that is closest to the agent is active. Either would work, but WTA was chosen simply because it was more efficient.

Landmarks can have connections between each other that give the agent knowledge about possible paths and the length of those. The black dashed lines in Figure 2 depict those connection vectors that inform the agent about the allocentric direction and distance of the adjacent landmarks. The yellow circles display important locations (which are also landmarks) that can serve as goals. Once a goal is set, the HPC searches for a sequence of vectors, on the cognitive map, to traverse from the currently active place cell to the place cell that gets activated for the goal location. It then applies vector calculations to that sequence in order to receive a goal vector (green arrow) that comprises the allocentric goal direction. Subsequently, this direction is encoded in a population of 360 allocentric goal direction cells, each tuned to a specific goal direction based on the agent’s location and sensitive to exactly 1° of encoded direction.

In other words if the goal direction vector points in allocentric direction of 36°, the AGD neuron sensitive to 36°becomes the most active one. Neighboring neurons show less activity whereas neurons far away are inhibited. This concept is commonly used and described in more detail by Sharp et al. (2001). To achieve a Gaussian shape of the signal, the excitation *e_i_* of neuron *i* at time *t* is calculated as follows:

(1) $$e_{i}\left(t\right)=\text{exp}\left(\frac{-{\left(\theta_{i}-\theta_{\mathrm{input}}\left(t\right)\right)}^{2}}{\sigma^{2}}\right)=G_{i}\left(\theta_{\mathrm{input}}\left(t\right),\sigma \right),$$

where θ*_i_* is the direction the neuron fires maximally, θ*_input_* is the input direction at time *t* (here allocentric goal direction), and σ a constant that is globally set to 10. We write *G_i_*(θ*_input_*(*t*), σ) in future equations.

This equation holds for **all** cell populations in our model, with the only variety in the input (e.g., head direction, allocentric goal direction, egocentric cue direction (ECD)). Note that after calculating the activity of a population, we add white Gaussian noise according to equation (2) and normalize it by dividing each value by the maximum of the population. This is essential since most cell equations have a coefficient that leads to values greater than 1.

(2) $$e_{i}\left(t\right)=e_{i}\left(t\right)+Z_{i},$$

where *Z_i_* is drawn from a zero-mean normal distribution *Z_i_* ~ *N*(*e_i_*(*t*), *N*). *N* describes the variance or noise and is set to 5. By adding noise, the cell populations more closely resemble the activity of biological cells, which requires the system to be robust enough to overcome this uncertainty.

The allocentric goal direction signal together with the sequence of landmarks (route) is subsequently forwarded to the RSC.

The landmarks are stored in the agent’s memory as Cartesian coordinates together with a connection matrix with distances for each connection. Currently, the system is designed for deployment on a robot on which the actual landmark locations are dynamically learned.

### 2.3 Retrosplenial Cortex

Compared to the HPC, the RSC is an under-researched brain area. However, recent investigations support its important role in spatial navigation (Vann et al., 2009). Its location in the brain as Brodmann areas 29 and 30 (Vogt, 1976) and the high density of connections to the HPC suggest its involvement in memory and navigation, which is supported by several studies with rodents (Vann et al., 2003; Vann and Aggleton, 2004). They show that RSC lesions strongly impair navigation. Those lesions have a bigger impact when animals are explicitly forced to switch between different navigational strategies (e.g., switching between allocentric and egocentric frame of reference and vice versa). The RSCs strong connectivity to the HPC and its bidirectional connectivity to the PPC suggest that a major function of the RSC in spatial frame transformation (Vann et al., 2009). This transformation is performed by shifting an allocentric frame (from HPC) by the current head direction, provided by head direction cells in the RSC (Cho and Sharp, 2001), to an egocentric frame and matching objects with information of the egocentric frame given by the PPC. The specifics of this transformation are an active area of research.

One possible explanation is provided by Alexander and Nitz (2015) in their work. In their spatial navigation experiments with rats, they recorded RSC activity “that is simultaneously sensitive to allocentric, route-centric, and egocentric frames of reference” (Alexander and Nitz, 2015). This study showed that the RSC comprises neurons that map egocentric, route-centric, or allocentric positions, and neurons that responded to multiple reference frames. It suggests that the RSC is in a unique position for spatial decision making. This process is assumed to take place in the RSC because of its access to all reference frames from HPC and PPC, as well as its connections to the prefrontal cortex (Vann et al., 2009; Nelson et al., 2014; Alexander and Nitz, 2015; Spiers and Barry, 2015). Navigational strategy selection can include the use of egocentric, allocentric, or route-centric frame, or a combination of these. It should be mentioned that other experiments indicate that the prefrontal cortex may also be responsible or at least involved in that strategy selection (Calton and Taube, 2009).

In our model, the RSC selects the frame of reference to deploy, which in turn influences the agent’s navigation strategy. The simulated RSC maintains a level of confidence for each frame, and uses this information to decide which frame of reference the model should use for navigation. Since the goal of this work is to show that the simulated agent can display similar behavior as real agents in actual experiments, the computation of each frame confidence level depends on the conducted experiment. We describe the actual method in detail for each experiment in the according section.

The model of the RSC is comprised of populations of head direction cells and receives the allocentric frame of reference from the HPC. It processes this information to perform a frame transformation from allocentric to egocentric representations.

**Figure 3:**
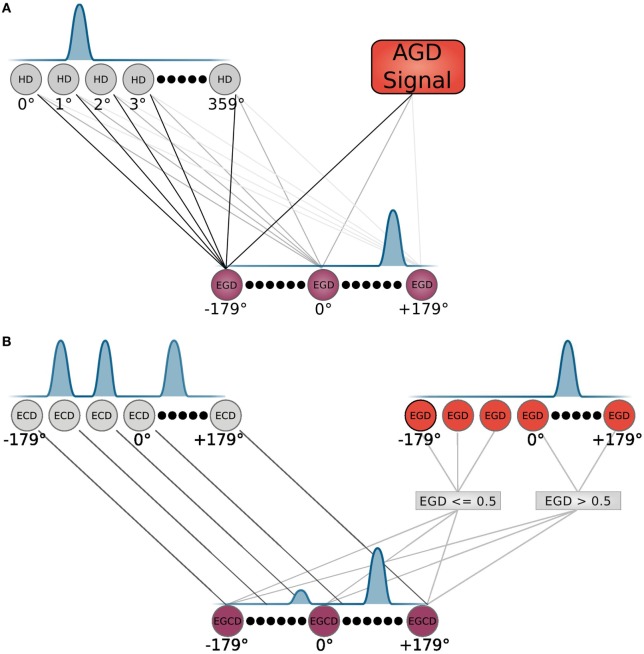
**Neuron modeling**. **(A)** HD and EGD neurons with their corresponding tuning and connections are depicted. **(B)** EGD, ECD, and EGCD neurons with their corresponding tuning. The EGD and ECD control the tuning of EGCD neurons via indicated connections. Connections represent an input to the equation that models the corresponding cell population.

The allocentric frame, i.e., the allocentric goal direction is transformed to an egocentric goal direction (EGD) by means of a population of head direction cells, as depicted in Figure 3A. These cells are constructed according to equation (1), with the input θ*_input_* given by the agent’s internal compass.

A population of 360 HD cells is maintained, driven by proprioceptive cues and the vestibular information, each tuned to a specific orientation of the agent’s head and sensitive to exactly 1° of the encoded direction.

**Figure 4:**
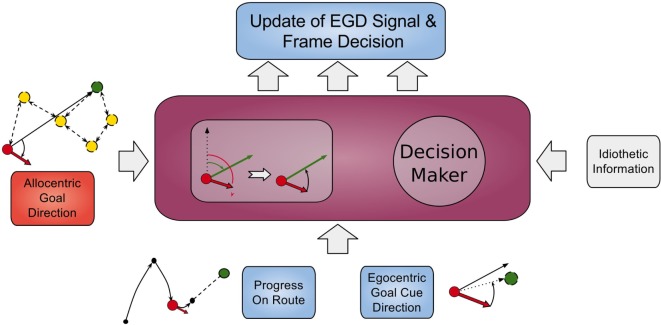
**Model of retrosplenial cortex**. The RSC receives AGD data, egocentric goal cue direction data, route progression, and idiothetic information as inputs and calculates the frame decision and an updated EGD signal.

Signals from those cells along with the AGD data are fed into a population of so called egocentric goal direction cells that are established using equation (1). However, for these cells, the input θ*_input_* is the difference between the head direction and allocentric direction. Thus, they indicate the direction of a goal in regard to the orientation of the head, e.g., an EGD signal of −80°means that the goal is located at 80°of the left hand side in the view field of the agent. We use *egd_i_*(*t*) to refer to EGD cell *i* at time *t*. This process is illustrated in Figure 4 in the smaller box inside the RSC (purple box).

#### 2.3.1 Decision Making and Confidence Levels

Determining a confidence value for a navigational strategy is rather complicated and strongly depends on the conducted experiment. We try to investigate which information is taken into account when choosing a strategy. Thus, we describe here the overall process that holds for every experiment.

A confidence level *c_i_* for a spatial navigation strategy *i* is a value in range [0, 1]. At the start of each trial, the initial value *c_i_*(0) for each strategy is set to 0.8 which shows that the agent is confident of his current position but it can still increase its confidence. In every time step, the previous value *c_i_*(*t* − 1) is automatically decreased by a decay value λ*_default_* = 0.02 that was chosen because of the length of the trials of the experiments. It has shown a good trade off between a too fast decreasing and a too slowly decreasing confidence value, which both led to a disfunction of the decision making process. It is described with the following formula:

(3) $$c_{i}\left(t\right)=c_{i}\left(t-1\right)*\left(1-\lambda_{k}\right),\;\text{where}\;k\in \left\{\mathrm{default},\mathrm{dist},\mathrm{stuck}\right\}.$$

This function represents an exponential decay process that can be found in several biological or physical processes. The reason for decreasing all confidence levels in each time step is the increase of uncertainty due to the agent’s movements and its sensors.

It is assumed that when an agent gets the “feeling” that it is getting closer to the goal, which might indicate that it is on a correct path, the agent increases its confidence in the used strategy by an increased value *u_default_* = 0.025. Again this value was chosen to ensure that the confidence levels increase by an appropriate rate. For that we simply add the value to the current confidence value:

(4) $$c_{i}\left(t\right)=c_{i}\left(t\right)+u_{k},\;\text{where}\;k\in \left\{\mathrm{default},\mathrm{ego},\mathrm{stuck},\mathrm{progress},\mathrm{direction}\right\}.$$

However, if the agent’s distance to the goal increases, it decreases the confidence of the applied strategy by means of the above described formula with a decay value of λ*_dist_* = 0.002. It appeared that this value works best with our environmental setups, since the agent is provided with enough time to decide whether the increase of distance to the goal is just to move around an object or because it chose an inappropriate strategy.

If the location of the agent stays the same for a longer time, it can be assumed that it got stuck and is not able to find a way to the goal using the current navigation strategy. Therefore, the confidence level of the current applied strategy is decreased by λ*_default_*, whereas the other levels are increased by *u_default_*.

After changing the confidence values, the frame of reference with the highest value will tend to be chosen. However, to soften the process and make it more biologically plausible a soft-max function is used:

(5) $$p_{i}\left(t\right)=\frac{e^{c_{i}\left(t\right)}}{\sum_{j=1}^{K}e^{c_{j}\left(t\right)}},$$

where *c_i_*(*t*) is the actual confidence value at time *t, p_i_*(*t*) the probability for confidence level *i* at time *t* and *K* the dimension of vector *p* [see Sutton and Barto (1998) for more details]. Assuming these values are ordered in an increasing manner, we can define corresponding intervals according to

(6) $$I_{i}=\left\{\begin{array}{cc} \left[0,p_{i}\left(t\right)\right],\; & \text{for}\;\underset{\forall i\in N}{\text{min}}\;p_{i}\left(t\right),\\ \left[p_{i}\left(t\right),1\right],\; & \text{for}\;\underset{\forall i\in N}{\text{max}}\;p_{i}\left(t\right),\\ \left[p_{i}\left(t\right),p_{i+1}\left(t\right)\right],\; & \text{otherwise.} \end{array}\right.$$

where *N* describes the number of navigational strategies. Subsequently, the RSC module draws one navigation strategy in regard to these intervals. The following equation describes that process:

(7) $$s=i\;\text{for}\;r\in I_{i},\forall i\in N,$$

where *s* describes the chosen strategy and *r* a uniformly distributed random value in the interval [0, 1].

This strategy decision together with the EGD signal is conveyed to the PPC where it subsequently determines the movement behavior of the agent.

### 2.4 Posterior Parietal Cortex

The PPC receives input from multiple sensory systems that includes the visual, auditory, vestibular, and somatosenory systems (Andersen and Gnadt, 1989). It uses this information to produce an egocentric frame of the local environment where the agent is currently located. This is done by extracting visual cues and determining their egocentric direction, i.e., determining the orientation in regard to the head of the agent and vice versa. Researchers have found neurons in the PPC that are specifically tuned for these cue directions (Snyder et al., 1998), so-called Egocentric Cue Direction cells. The head direction signal and the Egocentric Cue Direction signal both feed to so called “conjunctive cells” in the PPC (Wilber et al., 2014). A conjunctive cell is sensitive to a specific head and egocentric cue direction. Thereby they encode the angle the agent has to turn in order to face the cue. Wilber et al. (2014) showed that these cells predict movements of a rat and may be responsible for motor command signals that are sent to the motor cortex.

Lesions in the PPC indicate its major role in constructing the route-centric as well as egocentric frame (Committeri et al., 2004; Calton and Taube, 2009). Nitz (2012) tested rats in a loop environment that comprises five identically shaped squared spiral tracks. By means of that specially arranged environment, he was able to impose three different spatial reference frames on the rat and could record neural responses in the PPC for all three frames.

**Figure 5:**
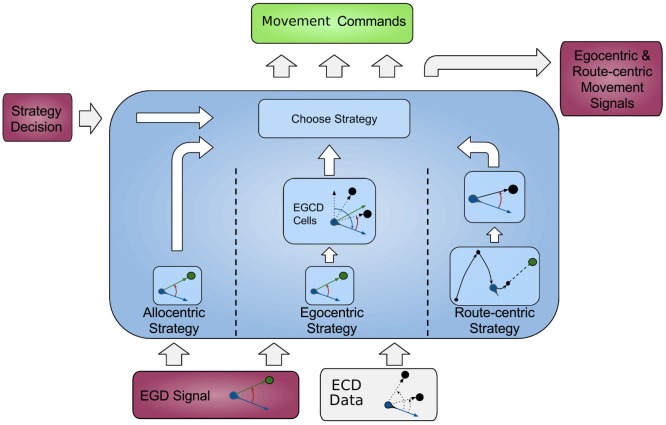
**Model of posterior parietal cortex**. The EGD and ECD signals are received as inputs and tune according movement cells depending on the conveyed strategy decision.

Based on these findings, the modeled PPC is responsible for converting the strategy decision given by the RSC into appropriate tuning of motor command cells that consist of three different neurons (left, right, and forward movement) and are connected to the motor cortex module. Depending on the activity of these cells, the motor cortex module determines executable motor commands. Different columns in Figure 5 depict navigation strategies not frames; however, these strategies rely on data of corresponding reference frames. It is necessary for the PPC to have access to all these navigation strategies since it is the link to the motor cortex.

The PPC also needs access to sensory information about the cues in the environment. Therefore, a sub-module of the PPC is constructed: the sensory module. This unit measures the distances and directions from the agent to perceptual cues. As described before the x and y-location of a cue is hard coded in a table and does not change over time. Therefore, a vector of tuples [distance *d_c_*(*t*) and direction θ*_c_*(*t*)] is calculated for every time step.

(8) $$\tilde{v}\left(t\right)=\left(\begin{array}{c} d_{c}\left(t\right)\\ \theta_{c}\left(t\right) \end{array}\right)=\left(\begin{array}{c} \sqrt{{\left(x_{a}\left(t\right)-x_{c}\right)}^{2}+\left(y_{a}\left(t\right)-y_{c}\right)}\\ \mathrm{co}s^{-1}\left(\frac{x_{c}-x_{a}\left(t\right)}{y_{c}-y_{a}\left(t\right)}\right)+\theta_{\mathrm{head}}\left(t\right) \end{array}.\right)$$

The distance *d_c_*(*t*) is the Euclidean distance from the current agent’s position (*x_a_*(*t*), *y_a_*(*t*)) to the location of cue (*x_c_, y_c_*). Whereas the egocentric direction θ*_c_*(*t*) is calculated by measuring the allocentric direction from the agent’s current position to the cue location and then shifted by the head direction θ*_head_*(*t*) of the agent.

In the following, we describe how the different navigation strategies are implemented in the PPC. All described cell types are based on equation (1). The decision to apply one of these strategies to calculate movement signals depends on the output of the RSC.

#### 2.4.1 Egocentric Strategy

The egocentric strategy in our model determines the action of the agent based on the activity of the movement cells, which received input from the egocentric cue direction cells and knowledge about perceptual cues in the vicinity of the agent.

An egocentric frame can be constructed from sensory inputs, such as auditory, touch, or vision, with the agent’s head direction as the main orientation. With that setting it is possible to determine directions and distances to cues, i.e., there is a big statue 40°to my left. The egocentric frame supports the orientation in small, visually manageable environments where all the relevant information can be expressed in relation to the subject’s position and within the extent of their current sensory experience. This property makes the egocentric frame impractical for larger areas, because in order to flawlessly apply the frame in an environment the agent has to keep track of all cues. However, the further the agent travels the more difficult it becomes to maintain all these cues and the egocentric frame thus becomes intractable over large spaces (Madl et al., 2015).

How the egocentric frame is applied in that strategy is shown in detail in Figure 3B. The top of the figure depicts two cell populations that serve as inputs for the egocentric goal cue direction (EGCD) cells: the EGD cells of the RSC and ECD cells from the sensory module, which carries information (distance and direction) about visible cues in the environment. These cells are tuned to specific cue directions in the visual field and show a Gaussian shape response for each cue. A special cell population called egocentric goal cue direction cells is maintained and initially receives input from the ECD signal. The EGD signal then modulates the EGCD population. That is, the cue signal with direction closest to the goal direction gets enhanced whereas all other signals get reduced. After that the EGCD population shows a strong response for the cue that is closest to the egocentric goal direction and weak responses for cues further away. This process refers to the egocentric navigation strategy and is illustrated in the middle column of the light blue box in Figure 5.

The excitation of ECD cell *i* at time *t* is described with

(9) $$\mathrm{ec}d_{i}\left(t\right)=\frac{D}{d_{c}\left(t\right)}*G_{i}\left(\theta_{c,\mathrm{in}}\left(t\right),\sigma \right),$$

where *d_c_*(*t*) is the distance from the agent to cue *c* at time *t*. Note that *d_c_*(*t*) cannot become 0, since the agent is not allowed to be at the same location as a cue. *D* is a constant set to 3.0 in order to relate the distance of the cue to the activation of the neuron. We chose this constant to facilitate our agent with a realistic range of vision. It can thereby recognize objects in a vista space but not beyond that. θ*_i_* is the direction the neuron fires maximally and θ*_c,in_* is the egocentric direction of cue *c* at time *t*. Note that this equation is based on equation (1).

Every neuron of the EGD cell population is connected to every EGCD neuron via a weight vector. This vector ensures that only cues close to the goal are considered and therefore lead to an excitement of the EGCD population, whereas cues far from the goal lead to an inhibition signal. A weight vector *w* for every neuron *i* of the 360 EGD neurons is calculated according to

(10) $$w_{i}\left(k\right)=G_{i}\left(k,\sigma \right),\text{ for}\;k=\left\{1,2...360\right\},$$

where θ*_i_* is the direction for that neuron *i* fires maximally. Note that here σ is set to 80, so that we have vector weights significantly larger than 0 for the complete range [1–360].

Using these weight vectors, the activity of an EGCD neuron *i* at time *t* is calculated as follows:

(11) $$\mathrm{egc}d_{i}\left(t\right)\;=\;{\mathrm{ecd}}_{i}\left(t\right)*\sum_{m=1}^{360}{\mathrm{egd}}_{m}\left(t\right)\;\left\{\begin{array}{cc} {}*w_{i}\left(m\right),\; & \;\;\text{if}\;{\mathrm{egd}}_{m}\left(t\right)>0.5,\\ {}*\left(w_{i}\left(m\right)-1\right),\; & \;\;\text{otherwise.} \end{array}\right.$$

The *ecd_i_*(*t*) signal of the egocentric cue direction cell *i* at time *t* is multiplied with the sum over every *egd_m_*(*t*) cell multiplied by the weight vector *w_i_*(*m*), if the EGD signal is strong enough (*egd_m_*(*t*) > 0.5) or with (*w_i_*(*m*) − 1) if the EGD signal is too weak. The latter one leads to an inhibition of the EGCD cells. The PPC applies this EGCD activity subsequently to determine the movement cell activity of the agent.

#### 2.4.2 Route-Centric Strategies

There are two possible ways for the agent to execute the route-centric strategy: (1) sequential egocentric, in which the route is a sequence of motor commands along a trajectory and (2) cue following, in which the route is made up of a sequence of landmarks.

#### 2.4.3 Route-Centric (Sequential Egocentric)

In the following starmaze experiment, the agent applies the sequential egocentric algorithm. This algorithm is based on a previously executed and stored sequence of motor commands with corresponding choice points, i.e., junctions. The sequence *S*(*j*) that leads to goal *j* consists of tuples (*l, m*) where *l* is a landmark and *m* is a corresponding movement.

(12) $$S\left(j\right)=\left\{\left(l_{i},m_{k}\right),\ldots \right\},\;l_{i}\in \text{all}\;\text{relevant}\;\text{landmarks}\;\text{for}\;\text{goal}\;j,m_{k}\in \left[-180,180\right].$$

The agent is programed to move straight until it encounters a landmark *l* of the sequence *s*(*j*), then the stored movement *m* is executed. This movement can either be a left or right turn, −180°or 180°, respectively. Once the agent has executed the movement and passed the landmark it continues to move straight. This is realized by feeding the movement directly into the movement cells. The PPC keeps track of the path progress and retrieves the corresponding motor command when approaching a choice-point. Thus it is a more developed version of the simple response strategy proposed by Packard and McGaugh (1996).

#### 2.4.4 Route-Centric (Cue Following)

Another route-centric strategy is cue following, which is depicted in the right column of Figure 5. This algorithm is based on a previously calculated sequence of cues *S_cues_* that has to be followed in order to reach the goal (in contrast to the previous strategy, where cues indicate a change in movements). The PPC keeps track of the path progress and determines the next cue to follow. It then checks for that cue in the visual field of the agent (top box of the column) and sends movement signals accordingly to the motor cortex to move in the direction of that cue. When the cue is reached, the succeeding cue is retrieved and the process starts again. This is done by identifying the goal cue in the cue vector, which comprises information about the distance and direction of that cue, and based on the information tuning the ECGD cells according to:

(13) $$\mathrm{egc}d_{i}\left(t\right)=G_{i}\left(\theta_{c}\left(t\right),\sigma \right),$$

where θ*_c_*(*t*) is the direction of the goal cue received from the goal vector. We do not consider the distance of the cue, since we assume that these cues are visible from every location of the environment. The PPC applies this EGCD activity subsequently to determine the movement cell activity of the agent.

Depending on the strategy decision *s* (see equation 7), the PPC utilizes different cell population data to generate movement cell activity. In case of egocentric and the route-centric strategy, the EGCD cells are directly connected to the movement cells so that for an object that is toward the left (right), the corresponding left (right) movement cells are active that lead to orientation toward the object. The activity of a movement cell *mv_i_* at time *t* can be calculated according to

(14) $$\begin{array}{ll} \begin{array}{rl} mv_{1}\left(t\right) & =\sum_{k=1}^{180}\mathrm{egc}d_{k}\left(t\right),\\ mv_{2}\left(t\right) & =\sum_{k=126}^{234}\mathrm{egc}d_{k}\left(t\right),\\ mv_{3}\left(t\right) & =\sum_{k=181}^{360}\mathrm{egc}d_{k}\left(t\right). \end{array} & \end{array}$$

On applying this equation it is guaranteed that the movement cell for left turns (*i* = 1) responses solely to activity of EGCD cells in range [1, 180], which is the left part of the visual field. Activity of EGCD cells in range [126, 234] leads to excitation of the movement cell (*i* = 2), responsible for straight movements. We chose these values since they represent a part of the visual field that can be considered as direct in front of the agent. The last movement cell (*i* = 3) is sensitive to activity in the upper half of EGCD cells (range [181, 360]) that represent the right part of the visual field.

#### 2.4.5 Allocentric Strategy

On the left hand side of Figure 5, the allocentric strategy is illustrated. Applying that strategy, the agent directly moves in the direction of the goal. It thereby follows the direction of the EGD signal that is previously calculated according to the allocentric direction of the goal. This is done by applying the EGD signal directly to determine the movement cell activity. Therefore, the EGCD cells *egcd_k_*(*t*) in equation (14) are substituted with the EGD cell data *egd_k_*(*t*) from the RSC. A correct and profound knowledge of the goal location and an exact estimate of the agent’s own position on the cognitive map are required in order to successfully navigate with that strategy.

### 2.5 Motor Cortex Module

The motor cortex is a brain area in mammals that plans and controls body movements. Each part of the body is represented within the motor cortex and controlled by that specific area. A neighboring area and a main input source for the motor cortex is the PPC. One of its functions is to transform multi-spatial information into actual motor commands that are then processed in the motor cortex to determine actual neuronal signals to move a muscle (Roland et al., 1980; Weinrich et al., 1984).

In our model these neuronal signals are combined in a velocity vector, consisting of velocity values for the left and right wheels of the agent that is used to move the agent in the virtual environment. This vector is calculated based on the conveyed movement cell activity from the PPC.

A velocity vector *v*(*t*) at time *t* is calculated as follows:

(15) $$v\left(t\right)=\left[mv_{2}\left(t\right)+mv_{3}\left(t\right),mv_{2}\left(t\right)+mv_{1}\left(t\right)\right],$$

where *mv_i_*(*t*) is the activity of movement cell *i* at time *t*. The velocity vector is then normalized by dividing it by 2 × *max*(*v*(*t*)), so that its values are in range [0, 0.5] and can be applied to calculate turning and forward movements of a simulated, as well as, a real agent.

### 2.6 Parameter Settings

Parameters were determined *a priori* to the actual experiment and are based on the parameters given in Section 2.3.1. See Table 1 for clarification. The left column contains values common for all experiments, whereas parameters in the middle column are only used in the starmaze experiment. The right column contains parameters solely for the vista space experiment and are described in Section 3.3.1.

**Table 1:** **Parameter values**.

All experiments	Starmaze experiment	Vista space experiment
λ*_default_* = 0.02	*u_ego_* = 0.05	*u_progress_* = 0.25
*u_default_* = 0.025		*u_direction_* = 0.005
λ*_dist_* = 0.002		λ*_stuck_* = 0.02
		*u_stuck_* = 0.01

## 3 Results

In this section, several simulation experiments are presented to show how the model can demonstrate comparable activity and behavior to empirical neural activity and behavioral findings. In particular, we conducted a blinking light experiment that shows how a special cell population can combine allocentric and egocentric frames, a starmaze experiment that investigates the usage of different navigation strategies based on allocentric and route-centric reference frames, and a vista space experiment where we showed that different frames of reference are crucial to successfully navigate in hierarchical environments.

### 3.1 Blinking Light Experiment

**Figure 6:**
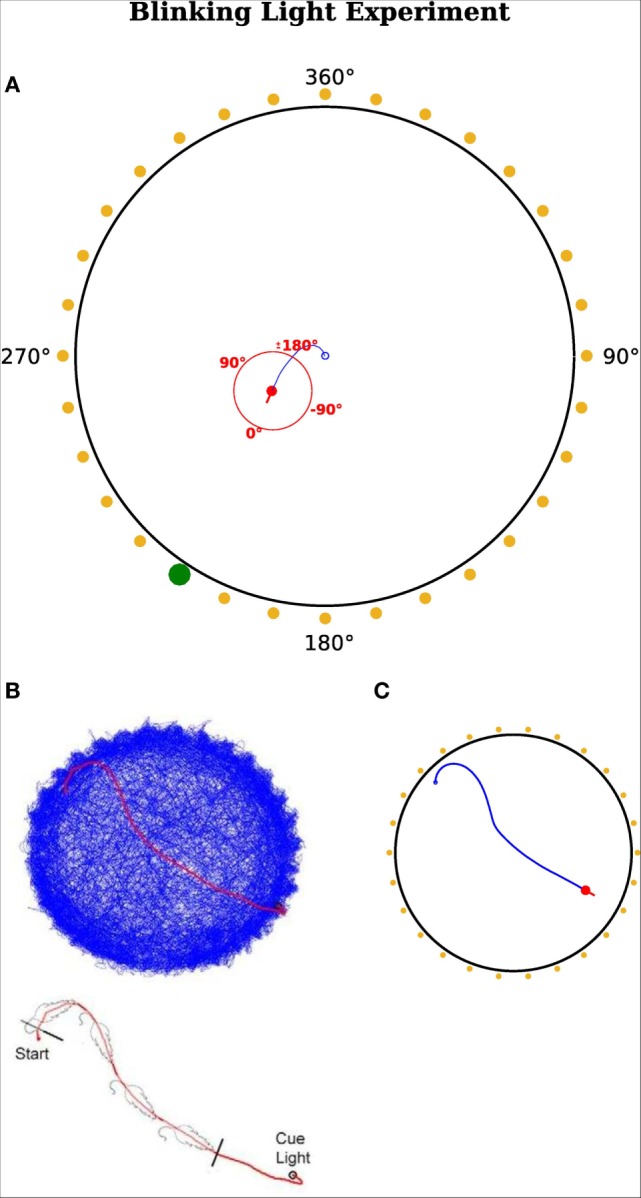
**Blinking light experiment**. **(A)** Red dot represents the agent with a red line indicating its head direction. Blue route shows the traveled path of the agent. Yellow points indicate a cue position. Yellow means this cue is not active and thus not visible for the agent. A green dot indicates it is active and the agent can sense it. The red circle illustrates the egocentric frame of the agent with corresponding degrees. 0°is always in front of the agent and turns accordingly. A cue at the right hand side of the visual field has a positive egocentric direction. The agent’s field of view is in range [−180°, 180°]. The fixed allocentric direction of the environment is implied with black numbers. **(B,C)** Agent’s movement comparison. **(B)** Results from Wilber et al. (2014). **(C)** Results from our simulation.

In this experiment, we reproduced the results of Wilber et al. (2014), who showed that neurons in the PPC encode egocentric and allocentric directions in terms of a rat’s heading and that the output of those neurons determines consecutive motor commands. Figure 6A shows the setup of their experimental paradigm. It composed of 32 cue lights surrounding a circular environment. There was only one actively blinking light at the time and a rat was trained to always move toward this blinking light. When the rat arrived in the vicinity of that cue, the light became inactive and another randomly chosen light started to blink.

Altogether, they used 8 rats and a sequence of up to 900 varying blinking lights in one session and measured 581 neurons in the PPC during the experiments.

Wilber et al. (2014) found conjunctive cell in the parietal cortex, which is a specially tuned cell that receives input from egocentric cue direction and head direction cells. Due to the conjunction of these two signals, the conjunctive cell is typically selective for one head direction and one specific egocentric cue direction.

We compared their measured neuron activity with activity in our model and showed that we can simulate signals of conjunctive, head direction, and egocentric cue direction cells on a cellular level. We assumed the agent to have a visual field of 360°, which means that the agent has egocentric cue direction cells in range [−180°, 180°]. An egocentric cue cell is tuned to a specific cue angle, so that it becomes active if a cue appears at the tuned angle in the visual field of the agent. Thereby the activity is independent of the allocentric direction of the agent. A head direction cell is sensitive to one specific allocentric orientation of the agent. The simulated conjunctive cells are connected to the motor cortex and thus can determine the movement of the agent.

Since there was no need to change navigational strategies, the decision making module of the RSC was inactive in this experiment. The agent used the egocentric strategy exclusively.

#### 3.1.1 Simulation and Results

We conducted two simulations in order to show that the model is able to produce similar neural activity for the agent’s egocentric cue direction and conjunctive cell population. Since Wilber et al. only recorded cells in the posterior partietal cortex and did not mention other brain areas, we disabled the HPC and RSC modules for this experiment and processed only cell populations of the PPC that is the egocentric goal direction, head direction and conjunctive cells. Moreover, it was reasonable to neglect these two areas here due to the egocentric characteristic of the experiment.

The PPC calculates the activity of the ECD cell population according to the visual information of cue locations it receives from the modeled sensory unit of the agent. A head direction signal is also maintained and together with the ECD signal fed into a population of conjunctive cells. These cells then exhibit tuning to a specific allocentric head direction and a specific egocentric cue direction. Thereby they combine an allocentric and egocentric frame of reference.

Subsequently, these cells produce a motor command signal which is conveyed to the motor cortex.

**Figure 7:**
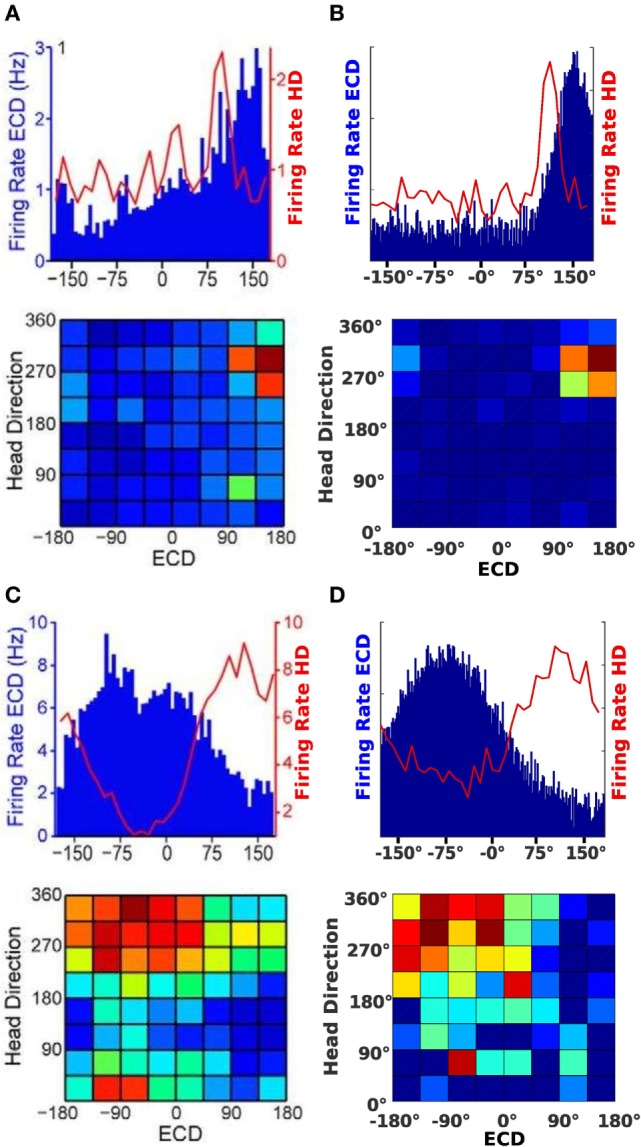
**Cell activity comparison**. **(A,C)** Result from data published by Wilber et al. (2014). **(B,D)** Result from our simulation experiments.

Figures 7A,C show the neural activity of rats at a specific time. The top plot in Figures 7A,B illustrates firing rates of a conjunctive cell for an egocentric direction of a cue (blue bars) and the rat’s head direction (red line). The bottom plots show a typical illustration for conjunctive cells. It plots head direction and ECD at the same time in one surface plot.

Since the plots in Figure 7A show a tuning to approximately 310°head direction and a +150°egocentric cue direction, we chose a simulated conjunctive cell with the same properties. The firing of this cell is plotted in Figure 7B. For the plots in Figure 7C, the head direction is approximately 350°and the egocentric cue direction firing indicates a cue at −90°. A corresponding cell of our model is plotted in Figure 7D.

Despite the similar neuronal activity, another focus was on simulating the actual behavior of the animal. Therefore, we compared the movement of the real rat with the trajectory of the simulated agent. Figures 6B,C illustrate the movement of the rat (Figure 6B) and the agent (Figure 6C). Figure 6B shows the recorded path of the rat in the environment. Figure 6C depicts the path of the simulated agent. Note that the simulated agent has similar orientation and approach behavior to that of the rat. Note that quantitative comparisons with the original experiments cannot be provided, because the authors did not make raw data available. However, both the behavioral and neural responses in Figures 6 and 7 are qualitatively similar.

### 3.2 Starmaze Experiment

The simulation described here is based on an experiment by Iglói et al. (2009), in which they tested humans in their use of two different navigation strategies in a virtual environment: an allocentric strategy based on allocentric cues in the environment and a sequential egocentric strategy that stores a temporal sequence of relations between movements and environmental choice points. They argued that humans have access to at least two different navigation strategies when navigating in a complex environment. In these experiments, Iglói et al. tested whether a person uses allocentric and sequential egocentric strategy initially or rather uses one strategy before the other. In the present simulation experiments, we challenged our model to produce the same results as described in the paper. Our main focus was thereby on the behavioral response of the model. We investigated how the confidence levels determine the behavior of the agent.

**Figure 8:**
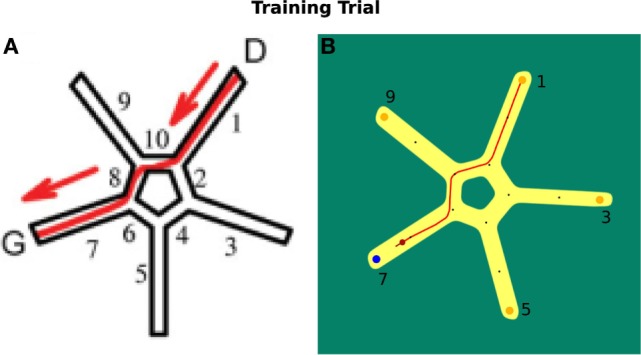
**Starmaze environment comparison**. **(A)** Virtual environment as described by Iglói et al. (2009). **(B)** Environment used in our simulation.

In Figure 8A, the virtual environment is shown. Figure 8B illustrates the environment we use in our simulation. Both are facilitated with allocentric cues.

In a training trial, the start position of a participant was located in alley 1 of the Starmaze. The person had to find the goal in alley 7. The test existed of 16 training trials and 5 probe tests that were not communicated to the participants. In a probe test, a person was located at alley 5 and tested whether he/she uses the sequential egocentric strategy, which results in finishing the trial in alley 1, or the allocentric strategy, which results in ending in alley 7. For the sequential egocentric strategy, the person solely applied the movement sequence learned in the training trials. For the allocentric strategy, the person relied on the cues to navigate to the goal location.

Our agent used the same navigation strategies, i.e., allocentric and route-centric (sequential egocentric) that were based on the previously introduced reference frames. In order to allow us to compare results from our simulation and results of human experiments, our agent could only choose between those two strategies. To decide which strategy is used next, the corresponding confidence levels were considered. The goal was to achieve similar results and behavior with our model as it is shown in the behavioral study of Iglói et al. (2009).

At the beginning of each trial in our simulation the applied frame was chosen probabilistically, with a chance of 25% for the allocentric frame and 75% for the sequential egocentric frame. This parameter was determined empirically in line with the results from Iglói et al. (2009).

To regulate the confidence value of the egocentric strategy, the egocentric goal cue direction was compared to the head direction of the agent and if both showed similar directions (in a range of ±10°) the confidence value was increased by *u_ego_* = 0.05.

#### 3.2.1 Simulation and Results

**Figure 9:**
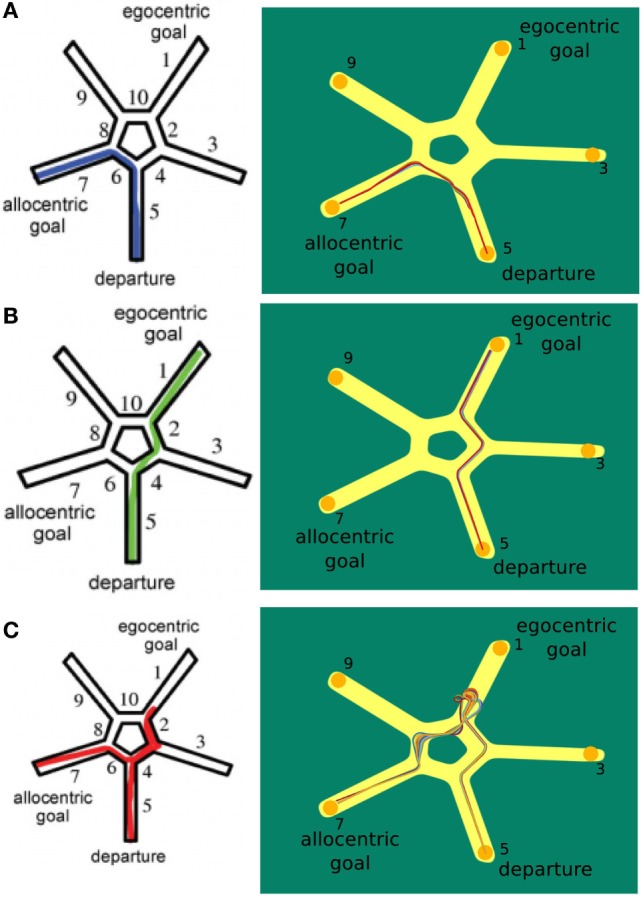
**Starmaze strategy comparison**. **(A)** Trials where the agent applied the allocentric strategy. **(B)** Trials where the agent solely used the egocentric strategy to navigate to the goal. **(C)** Trials depicting the use of a mixed strategy. Figures in left column are from Iglói et al. (2009).

In the first trial of our simulation, the agent learned the sequential egocentric sequence, moving from alley 1 to 7. After that it was placed in alley 5 and we investigated which strategy the agent would apply for reaching the goal. In total, we ran 130 trials and divided them into categories according to the strategy used. The allocentric strategy was used in 37 trials, the egocentric strategy was used in 59 trials, and the mixed strategy was used in 34 trials. Figure 9 shows these categories, left column depicts the results of Iglói et al. (2009) and right column displays our results.

The plots in Figure 9A display the application of the allocentric strategy. Both humans (left plot) and agent (right plot) exhibited similar trajectories. Their start location was in alley 5 and since they moved to the previously learned goal, the allocentric strategy was applied. Thereby, human and agent solely relied on allocentric cues and were able to navigate to the original goal in alley 7. To illustrate that the agent navigates by means of the allocentric strategy frequently, all 37 allocentric trials are depicted.

In contrast to that, Figure 9B illustrates the usage of sequential egocentric navigation. Starting in alley 5, the participants moved to the end of alley 1 which indicates that they were merely relying on idiothetic information and egocentric cues. This information was utilized to measure the progress on the egocentric movement sequence. The agent displayed similar trajectories over different trails (right hand side).

The trajectory for a mixed strategy is illustrated in Figure 9C. The person’s start location was in alley 5. He/she used the sequential egocentric strategy at the beginning which leads to the trajectory to alley 1. However, the underlying strategy changed, possibly because of a decrease in confidence, and the person navigated to the original goal in alley 7. The left column shows the trajectory of humans applying both strategies consecutively whereas the right column depicts multiple mixed trajectories of our agent.

**Figure 10:**
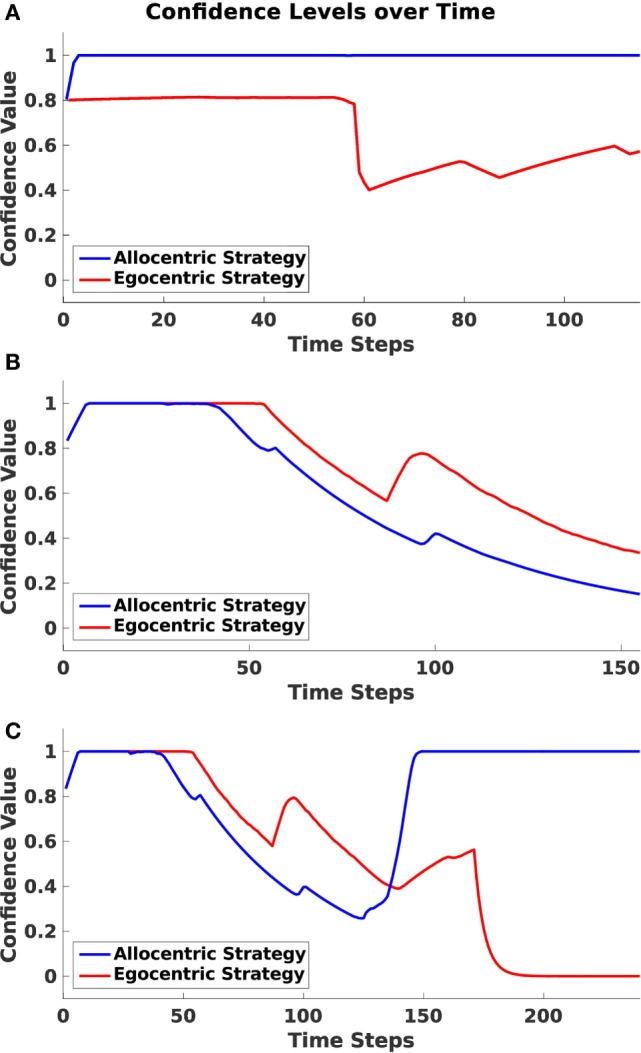
**Confidence levels over time**. Plots indicate the averaged allocentric and egocentric confidence levels over time over all runs. **(A)** Shows values for allocentric trials. **(B)** Shows values for sequential egocentric trials. **(C)** Shows values for mixed trials.

As mentioned before, the agent maintains confidence values for each strategy, which are determined by the similarity of the egocentric goal cue direction and the head direction of the agent (for more details, see Section 2.3.1). Based on these values, it decides which strategy has to be applied next. We measured those confidence values in every trial over time and categorized them according to whether the agent traversed the environment applying the allocentric, sequential egocentric, or mixed strategy. Figure 10 depicts these values.

In Figure 10A, values for an allocentric trial are drawn. One can identify a rapid decrease in the egocentric frame at time step 55. At this point, the agent has passed the first junction and has made a left turn instead of a right turn as proposed by the sequential egocentric strategy. By contrast, the allocentric confidence value remained on a steady high level during the entire trial.

During a sequential egocentric trial (Figure 10B), both strategies were maintained on the same level in the beginning. The agent started to lose confidence in the allocentric strategy when approaching the first junction at time step 50. Shortly after the junction then confidence value for the sequential egocentric strategy decreased as well and only increased once at time step 90 since the agent was able to successfully execute the previously learned sequence of turns.

In Figure 10C, confidence levels for the mixed strategy trial explicitly exhibit the strategy change. Until time step 125, the confidence values resembled the ones from the sequential egocentric trial, which is reasonable since the agent applied that strategy at the beginning. At time step 140, the allocentric confidence level increased rapidly. This corresponded with the time the agent turned around and moved in direction to the allocentric goal. However, it has to be mentioned that the agent had already switched from sequential egocentric to the allocentric strategy before the rapid increase of confidence. We explain that behavior in more detail in the next section. Once the agent switched to allocentric strategy, its confidence in that strategy increased and remained at a maximal level. In contrast to that, the sequential egocentric confidence value decreased and remained low until the end of the trial.

**Figure 11:**
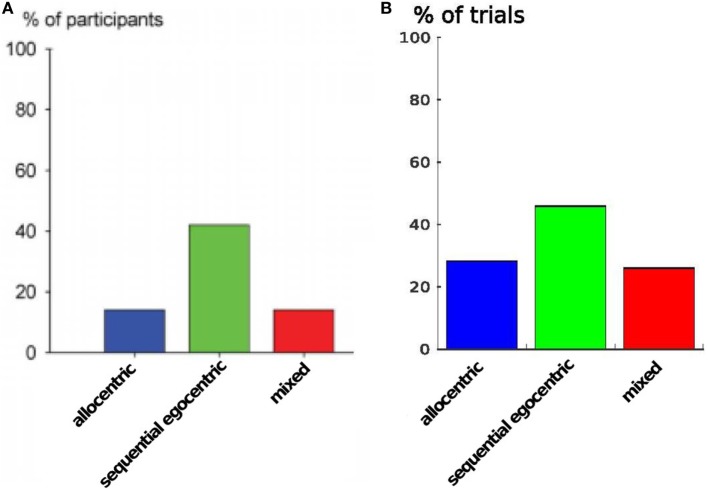
**Strategy usage over all runs**. **(A)** Results are from Iglói et al. (2009). **(B)** Results of our experiment.

Furthermore, to demonstrate that our model inherits the same behavioral properties we compare the strategy usage in Figure 11. The plot shows the percentages of different strategies in all participants: the sequential egocentric strategy was used the most with 42%, whereas the allocentric strategy as well as the mixed strategy was applied among 14% of the participants. In our simulation we achieved quite similar results. The sequential egocentric strategy usage was observed in 47% of the trials. Important fact is the ratio of the usage, it was the same as in real world experiments.

### 3.3 Vista Space Experiment

Research suggests that the representation of spatial memory is composed of different levels of detail, so that it can be used for a coarse-to-fine planning process. This means an agent, if located in large environments, can plan a route on a coarse level across different regions and is still able to navigate precisely to a given goal in a region. Based on this idea, Meilinger (2008) proposed a theoretical model that encodes visually perceptual information in so called *Vista Spaces* in which the subject can visually inspect the space (e.g., a room, a plaza). These spaces are connected to other vista space regions in order to efficiently navigate from a start to a goal location in a different region.

A similar system to the Vista Space was proposed by Wiener and Mallot (2003). In their experiment, they tested subjects in a virtual environment that was divided into different regions. Test subjects had to move from a starting location to a known goal location. Based on subjects traversed route in the virtual world, the researchers could make conclusions about the participant’s planning system. They stated that when planning a route in a well-known environment, subjects tend to apply a so called “Hierarchical Planning-hypothesis” (Wiener and Mallot, 2003). That is, test subjects plan route to a target region (where a goal is located) and move there as fast as possible. They then seek for the actual goal within that target region. This also corresponds to the model of Leiser and Zilbershatz (1989), where they suggested that in order to navigate to a goal in another region (vista space) an agent has to go through three steps: first, move from start position to the centroid of the region; second, plan the route from the start region’s centroid to the goal region’s centroid; and third, move from the goal region’s centroid to the goal.

In order to illustrate that our model can, by applying different frames of references, construct such a hierarchical reference memory system, we created experiments in accordance to these experimental data and their theoretical assumptions.

**Figure 12:**
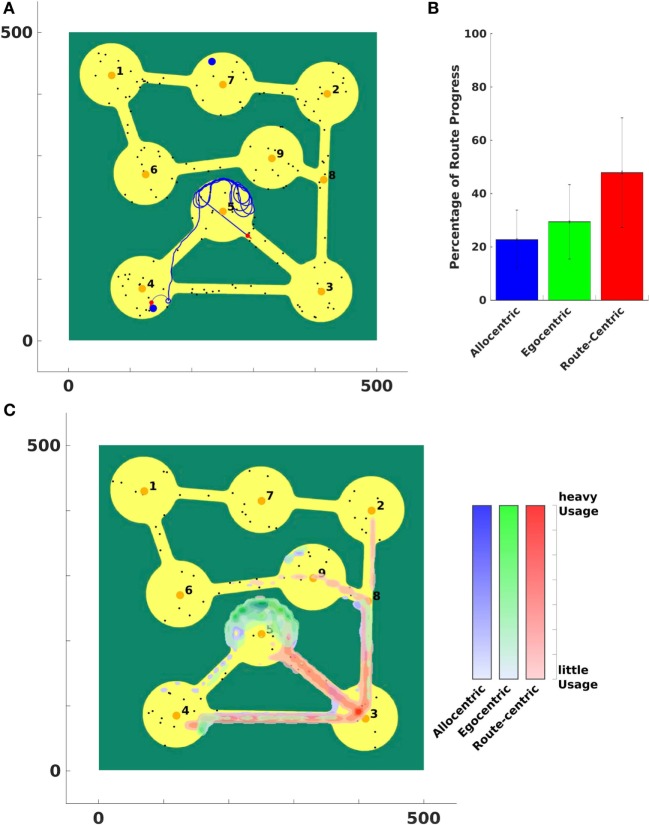
**Vista space first environment**. **(A)** The agent was stuck in region number 5 and is moving out of that region using route-centric navigation. **(B)** A percentual usage of strategies averaged over all trials. Black error bars indicate the standard deviation of over all trials. Panel **(C)** indicates where the agent used which strategy in the environment. It shows an average usage over all trials.

The experimental setup is shown in Figure 12A. Eight regions were connected with each other through narrow roads. Salient landmarks were located in a region, which served as goals of the agent. Each landmark belonged to exactly one region (the one closest to it). The agent’s start location was located in the bottom left of the environment (location 4). In each trial, a goal location was randomly chosen. The agent knew the location of the goal in its cognitive map and could thereby derive the allocentric goal direction (AGD). It also knew the topological structure of the environment and connections between regions that could be used to calculate a route using region centroids as landmarks. The route-centric frame comprised a sequence of those landmarks that had to be visited consecutively to reach the goal region.

#### 3.3.1 Confidence Levels

Besides the default calculations for confidence levels, the following conditions on confidence level changes were applied for this experiment. When the agent made progress along the route, it increased the confidence of the currently used strategy by *u_progress_* = 0.25. Progress on the route was determined if the next landmark in the sequence of landmarks of the route-centric frame was reached. This led to a certainty that the so far executed movements were correct and the agent was on its way to the goal.

To regulate the confidence of the egocentric strategy, the egocentric goal cue direction was compared to the head direction of the agent and if both pointed in similar direction (in a range of ±10°) the value was increased by *u_direction_* = 0.005.

Using the general confidence value calculations, as described above, the agent could sense if it got stuck in a circle and therefore lower the confidence of the currently applied strategy and increase the others. This was done by keeping track of the time spent near one place cell. If this time reached a threshold, the agent starts to decrease the confidence of the current strategy by decay value λ*_stuck_* = 0.02 and increases the others by *u_stuck_* = 0.01.

These parameters were determined *a priori* to the actual experiment and are based on the parameters given in Section *Decision Making & Confidence Levels*. See Table 1 for clarification.

#### 3.3.2 Simulation and Results

In each trial, the agent was placed at the start location (in the bottom left region of the environment) and a goal was chosen randomly from the previously defined cues. The agent then sought a path to the goal by applying different navigation strategies according to its confidence levels. It was capable of finding the goal in each trial. The cues in the environment were set at random locations and we run 80 trials for each environment.

In this representative example, the agent was tasked with traveling from a point in region 4 to a point in region 7. The agent could freely move and had a complete knowledge of the world’s topological representation, see Figure 12A.

Due to narrow paths between regions the agent had to apply route-centric navigation to efficiently travel from the start to a goal region to prevent the agent from getting stuck in a region. This behavior can be seen in Figure 12A. Allocentric and egocentric navigation strategies did not provide necessary information to lead the agent out of region 5.

In order to be able to measure the importance of a strategy and therefore a reference frame, we created a metric that determines the contribution of a strategy to a successful traversal from the start to the goal. We determined the shortest possible route from the agent’s position to the goal and summed up the steps, a strategy has been applied to progress on that route. Thereby it was possible to calculate the percentage contribution of a strategy on an agent’s route to the goal.

Figure 12B shows that metric for the first environment averaged over all trials. A strategy with a high value indicates that this strategy enabled the agent to significantly travel closer to the goal. By contrast, a low value indicates that the strategy is only of small support to get in vicinity of a goal.

To illustrate where in the environment a strategy was applied and to assess the performance of the agent, we plot the strategy usage in the world in Figure 12C. Blue indicates where the allocentric strategy is applied, green shows the usage of egocentric navigation, and red areas depict the application of route-centric navigation. Since many goals were located in the upper part of the world (regions 1, 2, 6, 7, and 9) the allocentric and egocentric strategy lead the agent to region 5 where it got stuck while applying the current strategy. After the confidence levels of those two strategies were low enough, the agent switched to route-centric navigation that guided it out of that region and away from the goal at the first place. This can be identified at the application of the route-centric navigation strategy. The agent started navigating with that strategy at region 5 (or 4) and continued all the way to region 3 and further on northward to the other more distant regions.

The agent was also able to use these strategies to flexibly cope with changes such as a route that is blocked. In Supplementary Material, we show two cases where the agent has to deal with a blocked path. In on case, it discovers the blockade, and in the other case, it is informed of this change, but in both cases, the agent successfully switches its navigation strategy to draw a new path toward the goal.

## 4 Discussion

The present paper introduces a model of the frames of reference and strategies used by animals and human while navigating through space. It demonstrates how these different frames are deployed under different navigational challenges. The agent’s behavior is guided by models of the HPC, RSC, and PPC. We make predictions as to how each of these areas contributes to navigation behavior. Specifically, we suggest that the HPC maintains an allocentric frame of reference, and the parietal cortex maintains a route-centric frame of reference, which gets converted into egocentric motor commands. The retrosplenial cortex decides which information to utilize when making decision by maintaining a confidence parameter in each for each of these reference frames. Although the idea of the brain calculating confidence levels is not plausible, the basal forebrain is a candidate for providing such a signal. It has been suggested that the basal forebrain, which has strong projections to the RSC, PC, and HPC, tracks expected uncertainty and that this might act as a confidence signal (Baxter and Chiba, 1999; Angela and Dayan, 2005; Avery et al., 2012). The simulation replicates a number of behavioral findings, ranging from neurophysiological experiments from an awake, behaving rat, to psychophysical experiments with humans in real and virtual environments. These results suggest that navigation strategies depend on the agent’s confidence in a particular reference frame and that the decision to rely on such information can fluidly change depending on sensory inputs. This has implications for flexible robot navigation. Note that we developed an abstract model of different brain regions and not all described cell populations might be biologically plausible. However, many model elements can be linked to cell populations in the brain. For example, we suggested that the basal forebrain might carry a confidence signal; furthermore, we speculate that the signal provided by the AGD population in our model could be related to goal sensitive place cells found in the HPC (Kobayashi et al., 2003).

### 4.1 Blinking Light

The blinking light experiment simulations show that our model is able to replicate conjunctive egocentric and head direction cell responses found in the PPC of awake, behaving rats (Wilber et al., 2014). These cells fire for a specific head direction in combination with an egocentric cue direction, thereby combining allocentric and egocentric frames of reference. In the blinking light experiments, the simulated agent was able to exhibit similar neuronal activity as in Wilber et al. (2014). We simulated the so called conjunctive cells in a virtual environment similar to the real one and demonstrated that their neural activity resembles the one described in the paper. Due to the characteristics of a conjunctive cell, we could also argue that the head direction and egocentric goal direction cells of our agent show biologically reasonable activity.

As shown in Figure 7, the modeled cells exhibit the same behavior as the conjunctive cells described by Wilber et al. (2014). This implies that our model also combines two reference frames. Since the agent utilizes the combined output of ECD and HD cells to determine the next movement of the agent, it implicitly applies signals of conjunctive cells to control future movements. Thereby we can also argue that the activity of HD and ECD cells in our model resemble actual recordings.

In order to imitate the behavior, the agent has to display similar movement characteristics. In Figures 6B,C, we show that the paths of the rat and the modeled agent resembles each other. The turning radius of both is similar. However, we are not able to explain why the rat’s path doesn’t show a straight line to the cue. We assume that it is an overshoot in the turning. For that reason and because we think that it does not effect the results, we do not model this behavior in our agent.

### 4.2 Starmaze

The starmaze experiments provided insights in how the egocentric sequential and allocentric strategy influence the agent’s behavior in an artificial starmaze environment. The agent produced actions similar to results of human experiments conducted by Iglói et al. (2009). Also the distribution of strategies applied by our agent was comparable to their results, except that we could not measure shifters due to our experimental setup. In their paper, Iglói et al. argue that both strategies are maintained simultaneously. However, they could not explain why participants suddenly changed their strategy and continued with that new strategy for the rest of the experiments. We could explain these switches by means of a confidence system that is continuously updated by the agent, based on the suggested movement of each strategy. Therefore, we can also support their argument for simultaneously maintained strategies, since only if an agent can compare strategies with each other it can decide which strategy might provide the best movements to succeed in terms of efficiency and/or time needed. For future improvements of the model, the confidence values could be used as a cost function and an optimization function could be applied in order to maximize confidences. Thereby the agent could be facilitated with a learning system that allows it to apply an optimal navigation strategy according to a given goal (shortest path, shortest travel duration, avoiding risks).

We demonstrated in the starmaze experiment that our agent exhibits the same behavioral properties as humans showed in real world experiments. The implemented navigation strategies calculated similar trajectories as humans did. The only difference can be seen in the mixed trajectory where the agent takes another route to the allocentric goal. It seems that when switching from the allocentric to the sequential egocentric strategy, the agent immediately senses the cue close to alley 9 and therefore moves in that direction. Moreover, we were able to tune the parameters of the confidence level system, so that the agent shifts from one strategy to the other even at the same time as humans did.

Iglói et al. conclude that both strategies are learned early and maintained simultaneously. We are able to support that argument and address it with our measured confidence levels. If both strategies are maintained simultaneously, the agent is able to construct confidence levels based on the suggested movements of each strategy. This would not be possible if an agent (human or modeled one) favored one strategy over another when unfamiliar with the task or environment.

The confidence approach is of special interest in the mixed strategy trials, since it explains why an agent switches from one strategy to the other. Due to the fact that both confidence values decrease with similar rate, the agent chooses another navigation behavior in order to increase its confidence value. We can observe this in Figure 10C.

Also the percentages of strategy use are similar to the results of Iglói et al. (2009). We can address the difference in the ratio to the fact that Iglói et al. consider shifters in their results. However, we were not able to measure the incidence of shifters in our environmental setup, since our model does not include a learning system over trials and consequently is not able to produce different results in different trials as human participants did in the original experiment. Therefore, shifters are irrelevant in our model and the allocentric and the mixed strategy are used more often.

### 4.3 Vista Space

In a last experiment, we investigated the concept of a hierarchical reference memory system of spaces and investigated whether our agent can take advantage of that. We were able to show that the agent’s overall performance increased when using the route-centric strategy between vista spaces. However, if a path is blocked the agent can benefit from the ability of applying other strategies. This made it flexible to react to sudden changes even without an implemented learning system. Based on our experiments, it seems reasonable that our agent implicitly used a hierarchical memory system of spaces.

For normal scenarios where the agent has complete knowledge about the environment, the simulation results and especially the extensive use of the route-centric strategy indicate that the most reliable strategy for the agent to find a goal is the route-centric, which was suggested by Wiener and Mallot (2003) and their hierarchical memory system. However, the route-centric strategy requires profound hierarchical knowledge of the environment and is more computational intensive than the other strategies, since the agent has to plan a route *a priori* using a route-planning algorithm. Note here that such an algorithm might not be plausible for biological agents.

In addition, if a path is suddenly blocked, as shown in environment 2 (see Supplementary Material) the agent has to either update its internal map of the environment and plan a new route, or apply a different strategy, as in Figures S1 and S2 in Supplementary Material. This situation illustrates the benefit of the other strategies since they use real-time information for navigating and can react to sudden changes. Whereas to successfully navigate with a route-centric strategy, the internal representation of the world has to be kept updated all time. If those strategies are not able to find a path to the goal after some time, the new information of the blocked road can be incorporated in the topological map, which subsequently allows the agent to plan a new, correct route.

The route centric strategy can then be applied to navigate the agent to the goal, as shown in Environment 3 (see Supplementary Material). Furthermore, it also indicates that the confidence level calculations are reasonable. Once the system notices that it cannot reach the goal using allocentric or egocentric navigation, it switches to the route-centric strategy, which subsequently leads it to the goal. This means that even though we didn’t program the confidence calculation system, it is still able to incorporate environmental changes.

These results are consistent with typical behavior of humans. In an experiment conducted by Wiener and Mallot (2003), participants learned an environment that could be distinguished in several different regions. After an initial learning phase, the persons were asked to move to a given goal. The researches observed that participants tried to travel to the target region (vista space) as fast as possible and, once arrived there, seek for the actual goal in that region. This showed that human are sensitive to regions in an environment and first plan a coarse trajectory (to a vista space) and afterward apply a finer planning to move to the actual goal (within the vista space). However, further experiments have to be conducted to investigate if solely a route-centric frame of reference is applied by humans in a connected vista space environment.

### 4.4 Comparison to Other Models of Navigation

In this section, we present some navigational models that are either investigating the concept of reference frames or relevant to our experiments in other ways.

The cognitive model of Byrne et al. (2007) includes the encoding of egocentric and allocentric maps as well as the transformation among them. Their primary focus is on the exact replication of the neural mechanisms for transformation and retrieval of spatial memory. Sensory input drives what they call boundary vector cells (BVC) (Barry et al., 2006), which enable translation from egocentric to allocentric and vice versa for retrieval and imagery of spatial information. Allocentric maps are stored in the HPC using neurons that are tuned to preferred allocentric directions and distances. These neurons correspond with the suggested BVCs. Egocentric maps are constructed by neurons that exhibit specific activity for a preferred distance and orientation of an object in the visual field of the agent. Thus, these neurons correspond to egocentric cue direction cells in our model. For each head direction, their model stores a separate population of these neurons that is connected to the corresponding egocentric representation in order to transform that egocentric map to the allocentric map. Because of the model’s biological similarity, it is able to simulate lesions in specific brain areas by disabling corresponding modules in the model. With that, they could perform experiments and compare their results to studies in human and rats as we did in our experiments. The model is able to store new representations of the environment, what separates their implementation from our navigational model. However, it lacks the ability to plan shortcuts or detours as we could show in the vista space experiment. Also we assume that for large environments, the necessary memory for storing each rotation of the egocentric map would quickly exceed possibilities for mobile agents. Nevertheless, it is a pioneer work for the investigation of translation of spatial frames of reference for navigation.

Another model that is worth mentioning here is the artificial neural network model proposed by Barrera and Weitzenfeld (2008) and Barrera et al. (2011). Their model is based on the neurophysiology of the brain and is therefore, as our model, biological plausible. However, their modeled architecture includes different areas of the brain, like the striatum for reinforcement learning, prelimbic cortex for storing a topological map of the environment, the entorhinal cortex for processing the current view of the animal, and the HPC for maintaining place cells (Barrera et al., 2011). They do not focus on modeling transformations of reference frames or even the frames itself. Nevertheless, their model is relevant since they first conducted particular navigation experiments with rats and then expose their robot to the same environmental setup and conducted the same experiments. From their achieved results they concluded that their model is neurologically plausible and can predict rodent’s spatial behavior. We were not able to conduct experiments with real rats, but instead we looked for behavioral and neurological results from human and rats, constructed the same experimental setups and exposed our simulated agent to those tasks. Based on our results, we could also conclude that our model is neurologically plausible and capable of predicting spatial behavior. In contrast to our simulation, they used a robot in a real environment with actual sensory input. We see this as a challenge for future work and plan to deploy our model on a real robot.

### 4.5 Limitations of Our Model

During the model design process, we had to make several critical design decisions and encountered different issues that led to limitations of our model.

One difficult task was to find a common and general definition of the term allocentric, route-centric, and egocentric. Even though these terms are frequently used, their definition may vary significantly from work to work. In order to overcome this issue, we adapted the most frequent definition of allocentric and egocentric frame of reference and defined the term of route-centric reference frame by taking into account the work of Wiener and Mallot (2003), Nitz (2006), and Calton and Taube (2009). However, those might differ from definition of other groups in that research field. The same issue holds for the definition of spatial navigation strategies. However, we describe each navigational strategy comprehensively and give references to the work of others where possible.

While constructing our model, we focused on investigating how and where frames of reference are established and maintained in the brain and especially how they can be applied by the agent to navigate to a goal. For that we modeled the underlying neuron population with a 1D Gaussian function, which is accurate enough for our purpose. However, to investigate these populations in more detail and to resemble actual neurological activity more accurate, we need a more proper model of neurons, such as a spiking model (Gerstner and Kistler, 2002). With that we might be able to examine the interaction of brain regions and the timing of when and where data are received by those regions. Also, to reduce the complexity of our model, we limited our simulation to the HPC, RSC, and the PC. However, we are aware that there exist other brain regions that contribute to spatial frames of reference and their application during navigation.

Future work should include the implementation of a learning mechanism in our model. Currently we do not implement any exploration algorithm, therefore the present system is based on previously stored and hard coded knowledge of the environment, like locations of landmarks and their connections between each other. This means that the agent is only capable of navigating in predefined maps, which is sufficient for the purpose of our current work. However, in order to deploy the model on a real robot and investigate its capabilities in real environments, the agent needs to acquire all this information on its own and incorporate it in its system. Therefore, we could imagine to implement a biologically inspired simultaneous localization and mapping system (SLAM) system or connect an existing one to our model in the future.

The HPCs ability to retrieve long-term memory and store short-term memory, especially for spatial navigation, is an important feature of cognitive models. In our model, the cognitive map refers to long-term memory and the egocentric cue information could be seen as short-term memory. However, we do not use these terms in our current system explicitly and do not model the storing process of short-term memory in the HPC. This learning process and the interaction and transformation of long-term and short-term memory might be implemented in future by expanding the functionality of the HPC.

The implementation of a learning system and the differentiation between long-term and short-term memory in our model would also facilitate comparison of our model to existing navigation models.

### 4.6 Advantages of Our Model

The concept of reference frames for spatial navigation is commonly used when animals have to navigate large, complex environments. By mimicking this capability, we might be able to facilitate a mobile agent with the same flexible and efficient navigation system. In combination with SLAM, our model could be helpful in situations where an agent has to navigate on its own in unknown or partly known environments. Especially, when an environment suddenly changes, then the agent, with its different navigation strategies, is flexible enough to find new paths to a goal. Whereas the SLAM system maintains an internal map for the agent, our model controls the agent’s movements and navigates it to a desired place.

Our implementation of the model is based on separated modules, each modeling a particular part of the brain with specified interfaces. Therefore, it is a straightforward process to substitute a module, e.g., with a more biologically plausible one, for example, an HPC that comprises a learning and memory system. This also enables us to simulate lesions in parts of our module to investigate spatial behavior and being able to reproduce even more real world experiments.

Another benefit of our navigation model is that it solely relies on a previously established cognitive map, visual, and idiothetic information. The model implicitly performs data fusion using that information. That is, it integrates visual input with idiothetic information in a cognitive map and retrieves information from that map to calculate movements.

Considering our results from a biological point of view, we could speculate that animals might have more than one route-centric navigation strategy (cue following, sequential egocentric) and that they might interfere with each other. To reduce error in navigation these strategies might be even combined eventually. Also, it could be imaginable that there are other specialized cell populations like neurons that are sensitive to an allocentric goal direction in animals’ navigational system.

## Author Contributions

TO contributed in model conception and design, experiment conduction, analysis and interpretation of data, and drafting manuscript. JK contributed in model conception and design, analysis and interpretation of data, and drafting manuscript. FR contributed in analysis and interpretation of data, drafting the manuscript, and critical review.

## Conflict of Interest Statement

The authors declare that the research was conducted in the absence of any commercial or financial relationships that could be construed as a potential conflict of interest.
