# Supplementary material for: A Computational Model for Spatial Navigation Based on Reference Frames in the Hippocampus, Retrosplenial Cortex, and Posterior Parietal Cortex
Source: Front Neurorobot. 2017 Feb 7;11:4. doi: 10.3389/fnbot.2017.00004 (PMC5293834; doi:10.3389/fnbot.2017.00004)
Supplement: Supplementary file 1 [file Data_Sheet_1.PDF]

Supplementary Material: A Computational Model for Spatial Navigation Based on Reference Frames in the Hippocampus, Retrosplenial Cortex and Posterior Parietal Cortex

Timo Oess [1,*], **Jeffrey L. Krichmar** [2] **and Florian Röhrbein** [1]
*Correspondence:
Timo Oess
timo.oess@tum.de

1 BACKGROUND ON NEUROSCIENCE AND BEHAVIORAL EXPERIMENTS

Three major brain regions are involved in constructing spatial frames of reference and transforming one into another. One of them is the hippocampus. It is located in the medial temporal lobe of the brain and is is necessary for the consolidation of information and the transformation from short-term to long-term memory. It also plays a major role in spatial navigation, particularly in establishing and maintaining a cognitive map of the environment (Tolman, 1948). The cognitive map is constructed in an allocentric representation and therefore all distances and orientations of objects in this map are encoded absolutely and are independent of the agent. In addition the cognitive map also encodes meta information for landmarks, objects or routes.

Due to its involvement in several major brain functions as memory consolidation, transformation from short-term memory to long-term memory and vice versa and its important role in spatial navigation and localization, the HPC has been greatly investigated over the last several decades. O'Keefe and Nadel stated in their work (O'keefe and Nadel, 1978) that the HPC established a cognitive map with the help of place cells. The cognitive map played a key role in every aspect of spatial navigation in larger environments. HPC lesions in rodents led to impairments in retrieval of spatial memory and as a consequence failure in navigational task (Morris et al., 1982; Dumont et al., 2015).

The most important feature of the HPC for our work is the encoded cognitive map. This map is enriched with numerous meta information for each landmark, path, cue or object and can be used for action planning in the environment (i.e., moving one's hand to grasp an object or calculating the shortest path from one place to another). Since the beginning of navigation research in rodents, it is assumed that rodents utilize the cognitive map in the HPC to retrieve and process vectors that encode spatial relations between entities in that cognitive map. Rodents use corresponding "vector-based calculations" to plan a route from one location to another (O'keefe and Nadel, 1978; Gallistel, 1990; McNaughton et al., 1996). Those calculations facilitate the agent to determine the allocentric direction to a goal from its current position. Using an allocentric direction and relations between objects in the environment an allocentric frame is constructed and maintained in the hippocampus.

The HPC is connected to the retrosplenial cortex. This functional unit is located in Brodmann areas 29 and 30 and is involved in several cognitive processes as scene translation, navigation, episodic memory and imagine future events (Brodmann, 2007; Aggleton and Vann, 2004; Vann and Aggleton, 2002; Cooper and Mizumori, 2001; Osawa et al., 2006; Vann et al., 2009). The RSC's strong connectivity to the HPC and its bidirectional connectivity to the PPC suggest a major function in spatial frame transformation. Several studies with rodents support this suggestion (Vann et al., 2003; Vann and Aggleton, 2004; Vann et al., 2009). They show that RSC lesions strongly impair navigation. Those lesions have a bigger impact when animals are explicitly forced to switch between different navigational strategies (e.g., switching between allocentric and egocentric frame of reference and vice versa).

How this transformation is accomplished in detail is an active area of research. One possible explanation is provided by Alexander & Nitz in their work (Alexander and Nitz, 2015). In their spatial navigation experiments with rats, they recorded RSC activity "that is simultaneously sensitive to allocentric, route-centric and egocentric frames of reference" (Alexander and Nitz, 2015). This study showed that the RSC comprises of neurons that map egocentric, route-centric, or allocentric positions, and neurons that responded to multiple reference frames. It also suggests that the RSC is in a unique position for spatial decision making.

This decision making process is assumed to take place in the RSC because of its access to all reference frames from HPC and PPC, as well as its connections to the prefrontal cortex (Spiers and Barry, 2015; Vann et al., 2009; Alexander and Nitz, 2015; Nelson et al., 2014). Navigational strategy selection can include the use of egocentric, allocentric or route-centric frame or a combination of those. It should be mentioned that other experiments indicate that the prefrontal cortex may also be responsible or at least involved in that strategy selection (Calton and Taube, 2009).

The posterior parietal cortex is a large cortical area thought to be responsible for many different tasks such as perception of pain (Witting et al., 2001), use of episodic memory (Cabeza et al., 2008), planning and controlling of movements (Andersen et al., 1997; Buneo and Andersen, 2006) and establishing of an egocentric reference frame (Stein, 1992; Wilber et al., 2014; Calton and Taube, 2009). It consists of Brodmann area 5 and 7 and receives input from multiple sensory systems, that includes the visual, auditory, vestibular, and somatosenory systems (Andersen and Gnadt, 1989). It uses this information to produce an egocentric frame of the local environment the agent is currently located in. This is done by extracting visual cues and determining their egocentric direction i.e. determining the orientation in regard of the head of the agent. Researchers have found neurons in the PPC that are specifically tuned for these cue directions (Snyder et al., 1998). Signals from these neurons and head direction signal are both feed to so called "conjunctive cells" in the PPC (Wilber et al., 2014). A conjunctive cell is sensitive to a specific head and direction. Thereby they encode the angle the agent has to turn in order to face the cue. Wilber et al. show in their paper (Wilber et al., 2014) that these cells predict movements of a rat and may be responsible for motor command signals that are sent to the motor cortex.

Lesions in the PPC indicate its major role in constructing the route-centric as well as egocentric frame (Calton and Taube, 2009; Committeri et al., 2004). Nitz (Nitz, 2012) tested rats in a loop environment that comprises five identically shaped squared spiral tracks. By means of that specially arranged environment he was able to impose three different spatial reference frames on the rat and could record neural responses in the PPC for all three frames.

2 VISTA SPACE ENVIRONMENT

In each trial the agent was placed at the start location (in the bottom left region of the environment) and a goal was chosen randomly from the previously defined cues. The agent then sought a path to the goal by applying different navigation strategies according to its confidence levels. It was capable of finding the goal in each trial. The cues in the environment were set at random locations and we run 80 trials for each environment.

2.1 Environment 2: blocked path, uninformed agent

In this environment, the path between region 3 and 2 is blocked without informing the agent's topological representation about that. Therefore it had to use different navigation strategies in order to reach the goal, see figure S1.

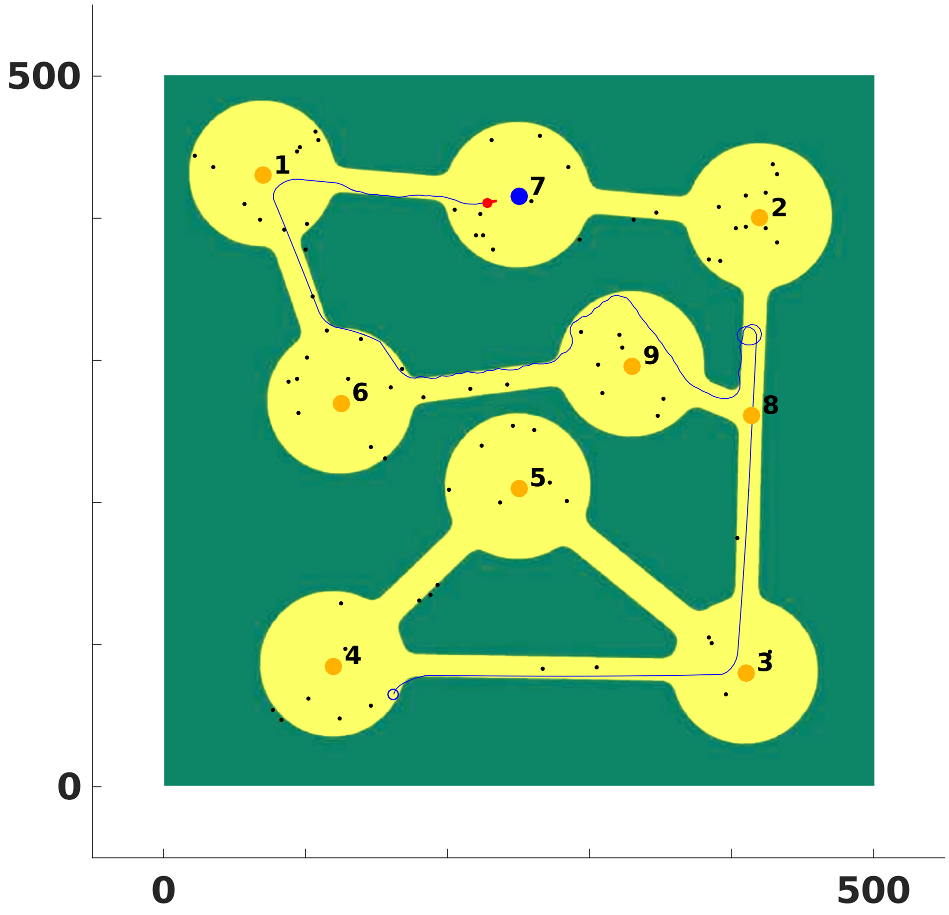

Figure S1. Vista Space Second Environment. Agent applied route-centric navigation strategy until it encountered a blocked path. It was stuck there for a while until it changed the strategy to navigate on a new path to the goal.

The purpose of this environment was to show that the agent was capable of navigating correctly and efficiently only if it maintains a correct hierarchical and topological representation. Therefore the path between region 2 and 3 was blocked and without informing the agent. That is, the stored topological map was not updated with the environmental change, which led to a wrong assumption of the route-centric frame and therefore to a failing route-centric navigation. Navigating with that strategy, the agent got stuck

at landmark 8 since the route-centric sequence determined landmark 2 as the next one to visit on the way to the goal, see figure S1. The agent had to switch to another navigation strategy in order to get out of the dead-end.

Figure S2 illustrates that the agent did exactly that. It encountered the blocked road above landmark 8 and after realizing that it got stuck, due to the steady activity of only the same landmark, the agent tended to switch to egocentric navigation in order to move out of the dead-end road and in direction of the goal region. Since the knowledge of the route-centric frame was still incorrect and therefore the confidence for that strategy did not rise again, the agent did not switch back to it but kept using the allocentric or egocentric strategy to succeed.

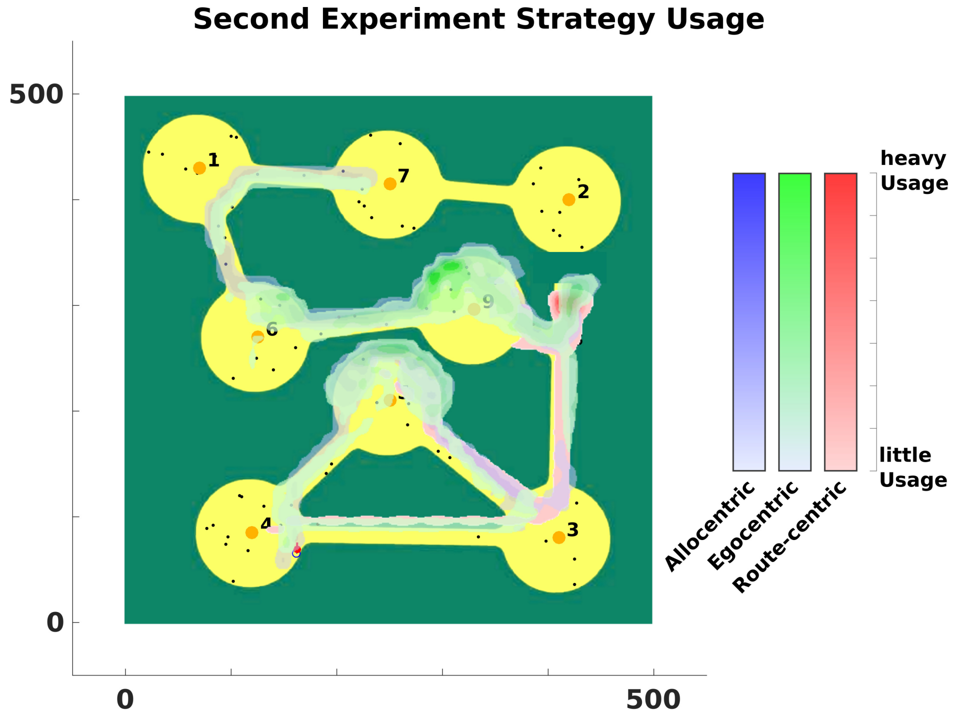

Figure S2. Strategy Usage in Second Environment. Indicates where the agent used which strategy in the environment. It shows an average usage over all trials.

In contrast to the previous experiment, here, the egocentric strategy indicated most progress on the way to the goal (see S3). This resulted from the application of that strategy starting from the blocked road continuously to the goal in region 7. The second most progressing strategy was the route-centric one since it was usually applied from the start region until the area closed to the blocked road.

2.2 Environment 3: blocked path, informed agent

This environment comprises a blocked path as well, this time between region 6 and 9. It differs from environment 2 in a sense that the agent was only informed about that blocked path after a specific amount of time has passed and could thereby update its internal topological representation, subsequently it could apply route-centric strategy to navigate to the goal. This is shown in figure S4.

To investigate how the agent reacts to a suddenly blocked path when it is informed about that - here we assumed that the agent is capable of learning a new topological representation - we constructed the environment shown in S4. The agent was confronted with a suddenly blocked path between region 9

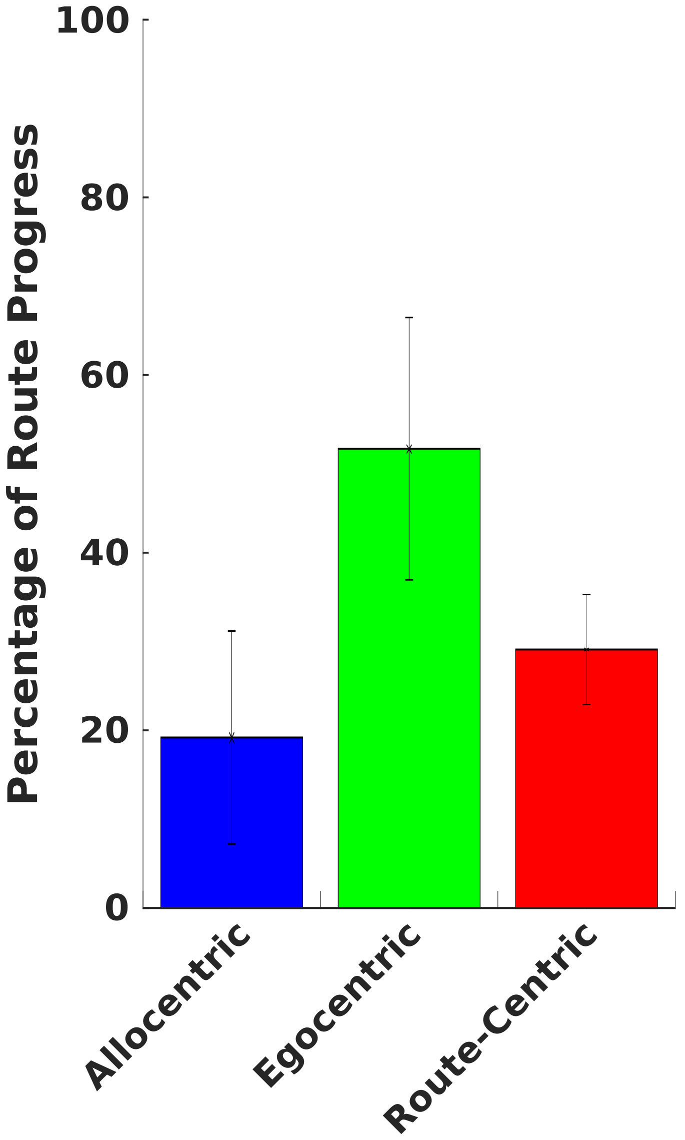

Figure S3. Progress per Strategy in Second Environment. A percentual usage of strategies averaged over all trials. Black error bars indicate the standard deviation over all trials.

and 6. It tried to find a way round, yet the route-centric navigation strategy constantly commanded it to move in the direction of the blocked path. Therefore the agent got stuck at blockage. However, in contrast to the previous environments, after some time the stored topological map was updated according to the environmental changes. This enabled the agent to calculate a new route and led subsequently to a successful navigation to the goal in region 6, utilizing route-centric navigation strategy. Its purpose was to emphasize that the agent - given a correct topological representation - is flexible to plan new routes when completely stuck in the environment.

As one can identify in figure S5, the agent mainly used the route-centric navigation to execute its movements. In an early stage this led to the blocked road between region 9 and 6. The agent stayed there and tried to find a way to the goal utilizing any available strategy (overlapping colors of blue, green and red). After a specific time the agent incorporated the new information about the blocked road in its topological map and was therefore able to plan a new route around the obstacle. It then navigated on the route using route-centric navigation.

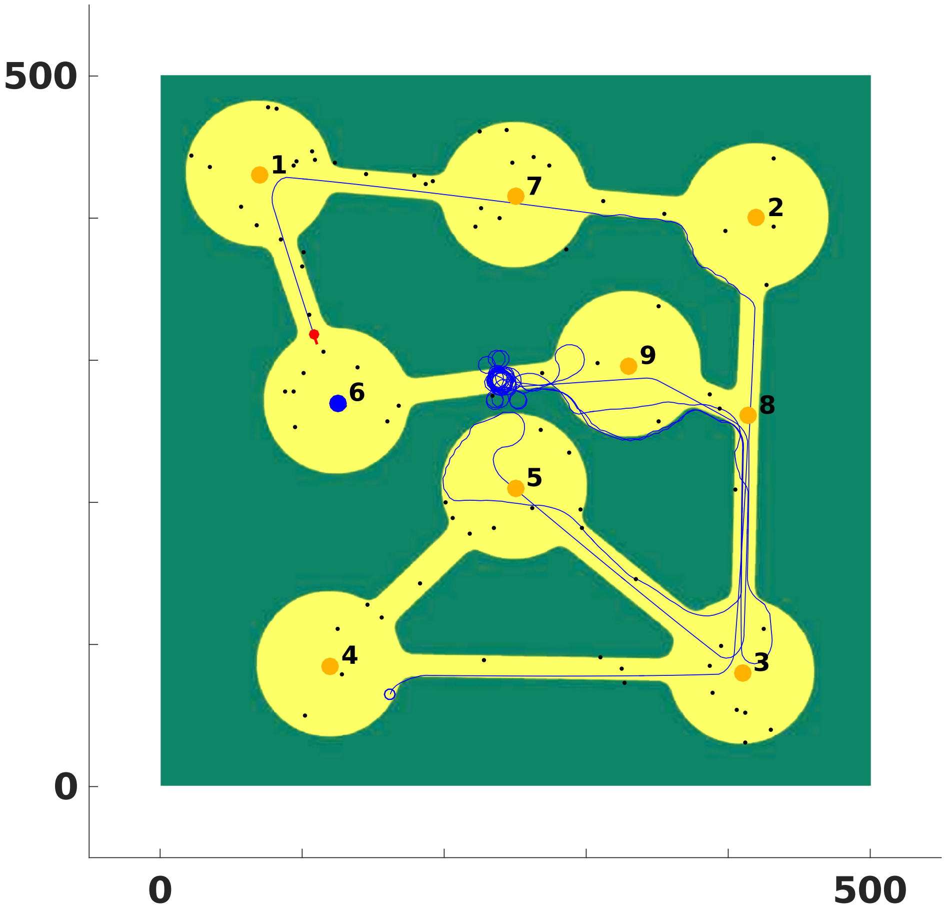

Figure S4. Vista Space Third Environment. Agent moved on correct path till it encountered a blocked route and was stuck. After a while the agent is informed about the blocked route and it incorporated the recently received information in its cognitive map of the environment. Subsequently it applied the route-centric strategy to move on a new route to the goal.

Figure S6 **A** shows that the distance to the goal decreased significantly while navigating with route-centric strategy, whereas the allocentric and egocentric navigation strategies contributed only less to get in vicinity of the goal. This results from the fact that the agent was unable to navigate to the goal applying an egocentric or allocentric strategy. Therefore the route-centric navigation was utilized more often.

REFERENCES

Aggleton, J. P. and Vann, S. D. (2004). Testing the importance of the retrosplenial navigation system: lesion size but not strain matters: a reply to harker and whishaw. *Neuroscience & Biobehavioral Reviews* 28, 525–531

Alexander, A. S. and Nitz, D. A. (2015). Retrosplenial cortex maps the conjunction of internal and external spaces. *Nature neuroscience* 18, 1143–1151

Andersen, R. A. and Gnadt, J. W. (1989). Posterior parietal cortex. *Rev Oculomot Res* 3, 315–335

Andersen, R. A., Snyder, L. H., Bradley, D. C., and Xing, J. (1997). Multimodal representation of space in the posterior parietal cortex and its use in planning movements. *Annual review of neuroscience* 20, 303–330

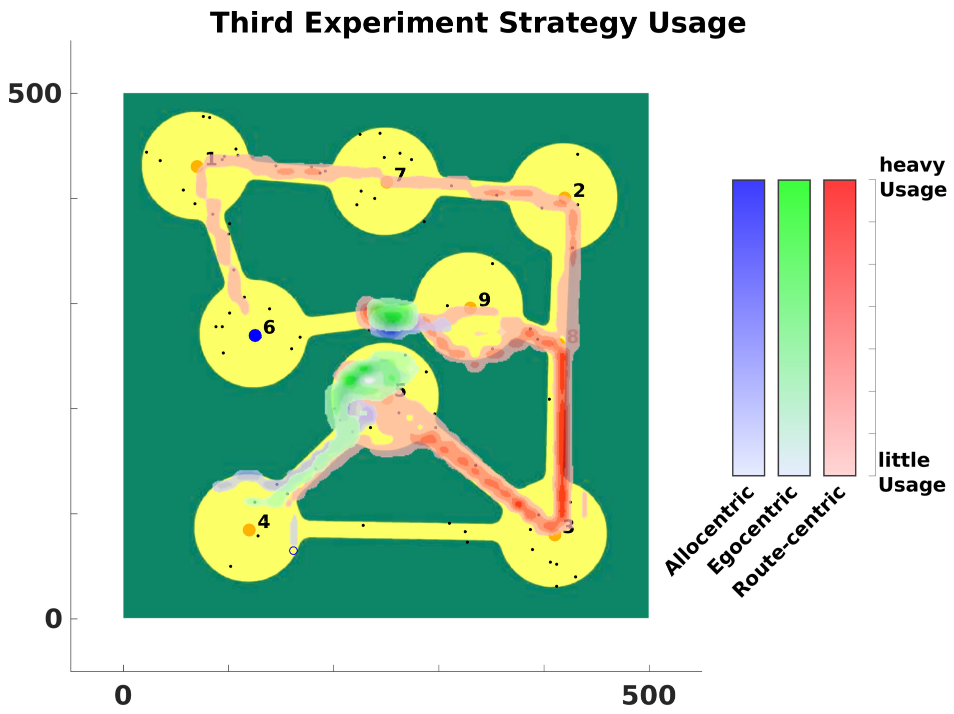

Figure S5. Strategy Usage in Third Environment. Indicates where the agent used which strategy in the environment. It shows an average usage over all trials.

Brodmann, K. (2007). *Brodmann's: Localisation in the Cerebral Cortex* (Springer Science & Business Media)

Buneo, C. A. and Andersen, R. A. (2006). The posterior parietal cortex: sensorimotor interface for the planning and online control of visually guided movements. *Neuropsychologia* 44, 2594–2606

Cabeza, R., Ciaramelli, E., Olson, I. R., and Moscovitch, M. (2008). The parietal cortex and episodic memory: an attentional account. *Nature Reviews Neuroscience* 9, 613–625

Calton, J. L. and Taube, J. S. (2009). Where am i and how will i get there from here? a role for posterior parietal cortex in the integration of spatial information and route planning. *Neurobiology of learning and memory* 91, 186–196

Committeri, G., Galati, G., Paradis, A.-L., Pizzamiglio, L., Berthoz, A., and LeBihan, D. (2004). Reference frames for spatial cognition: different brain areas are involved in viewer-, object-, and landmark-centered judgments about object location. *Journal of Cognitive Neuroscience* 16, 1517–1535

Cooper, B. G. and Mizumori, S. J. (2001). Temporary inactivation of the retrosplenial cortex causes a transient reorganization of spatial coding in the hippocampus. *The Journal of Neuroscience* 21, 3986–4001

Dumont, J. R., Amin, E., Wright, N. F., Dillingham, C. M., and Aggleton, J. P. (2015). The impact of fornix lesions in rats on spatial learning tasks sensitive to anterior thalamic and hippocampal damage. *Behavioural brain research* 278, 360–374

Gallistel, C. R. (1990). *The organization of learning* (MIT press Cambridge, MA)

McNaughton, B., Barnes, C., Gerrard, J., Gothard, K., Jung, M., Knierim, J., et al. (1996). Deciphering the hippocampal polyglot: the hippocampus as a path integration system. *Journal of Experimental Biology* 199, 173–185

Morris, R., Garrud, P., Rawlins, J., and O'Keefe, J. (1982). Place navigation impaired in rats with hippocampal lesions. *Nature* 297, 681–683

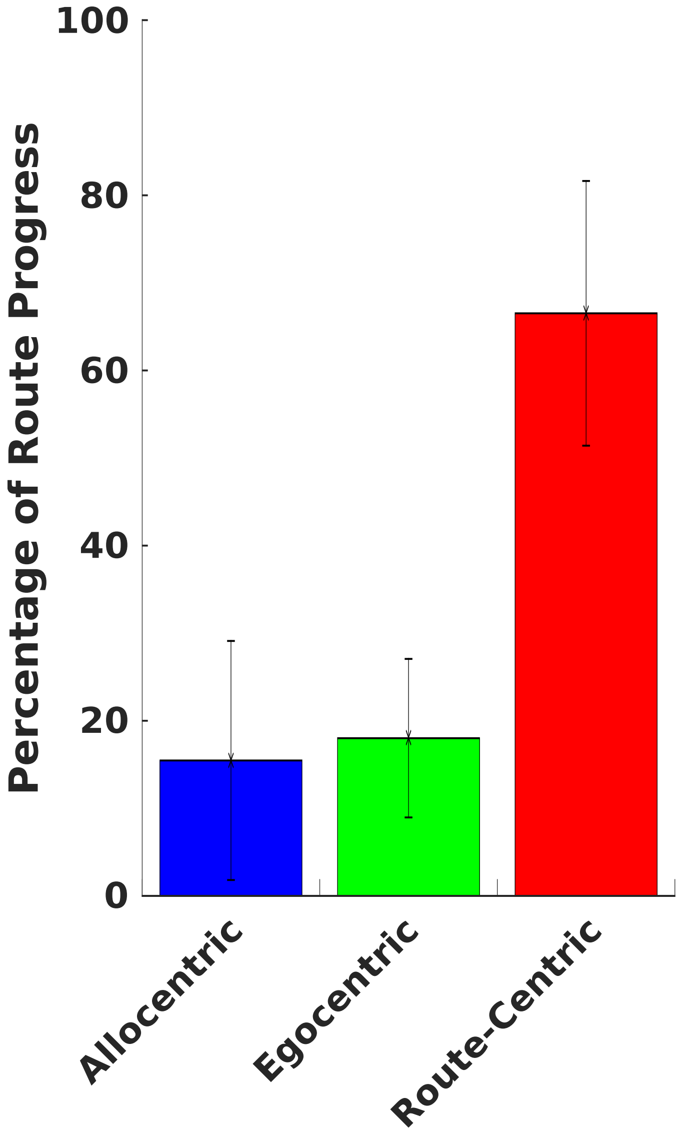

Figure S6. Progress per Strategy in Third Environment. A percentual usage of strategies averaged over all trials. Black error bars indicate the standard deviation over all trials.

Nelson, A. J., Hindley, E. L., Haddon, J. E., Vann, S. D., and Aggleton, J. P. (2014). A novel role for the rat retrosplenial cortex in cognitive control. *Learning & memory* 21, 90–97

Nitz, D. A. (2012). Spaces within spaces: rat parietal cortex neurons register position across three reference frames. *Nature neuroscience* 15, 1365–1367

O'keefe, J. and Nadel, L. (1978). *The hippocampus as a cognitive map*, vol. 3 (Clarendon Press Oxford)

Osawa, A., Maeshima, S., Kubo, K., and Itakura, T. (2006). Neuropsychological deficits associated with a tumour in the posterior corpus callosum: a report of two cases. *Brain Injury* 20, 673–676

Snyder, L. H., Grieve, K. L., Brotchie, P., and Andersen, R. A. (1998). Separate body-and world-referenced representations of visual space in parietal cortex. *Nature* 394, 887–891

Spiers, H. J. and Barry, C. (2015). Neural systems supporting navigation. *Current opinion in behavioral sciences* 1, 47–55

Stein, J. F. (1992). The representation of egocentric space in the posterior parietal cortex. *Behavioral and Brain Sciences* 15, 691–700

Tolman, E. C. (1948). Cognitive maps in rats and men. *Psychological review* 55, 189

Vann, S. D. and Aggleton, J. P. (2002). Extensive cytotoxic lesions of the rat retrosplenial cortex reveal consistent deficits on tasks that tax allocentric spatial memory. *Behavioral neuroscience* 116, 85

Vann, S. D. and Aggleton, J. P. (2004). Testing the importance of the retrosplenial guidance system: effects of different sized retrosplenial cortex lesions on heading direction and spatial working memory. *Behavioural brain research* 155, 97–108

Vann, S. D., Aggleton, J. P., and Maguire, E. A. (2009). What does the retrosplenial cortex do? *Nature Reviews Neuroscience* 10, 792–802

Vann, S. D., Wilton, L. K., Muir, J. L., and Aggleton, J. P. (2003). Testing the importance of the caudal retrosplenial cortex for spatial memory in rats. *Behavioural brain research* 140, 107–118

Wilber, A. A., Clark, B. J., Forster, T. C., Tatsuno, M., and McNaughton, B. L. (2014). Interaction of egocentric and world-centered reference frames in the rat posterior parietal cortex. *The Journal of Neuroscience* 34, 5431–5446

Witting, N., Kupers, R., Svensson, P., Arendt-Nielsen, L., Gjedde, A., and Jensen, T. S. (2001). Experimental brush-evoked allodynia activates posterior parietal cortex. *Neurology* 57, 1817–1824
